# When Does Sharing Stigmatize? Saving Money (vs. Seeking Variety) Through Access-Based Consumption

**DOI:** 10.3389/fpsyg.2021.778290

**Published:** 2021-11-18

**Authors:** Yang Jenny Guo, Cait Lamberton

**Affiliations:** ^1^Joseph M. Katz Graduate School of Business, University of Pittsburgh, Pittsburgh, PA, United States; ^2^The Wharton School, University of Pennsylvania, Philadelphia, PA, United States

**Keywords:** access-based consumption, financial constraints, happiness, communications, resource affordability, resource variety

## Abstract

Access-based services allow financially-constrained individuals to consume a variety of goods and services without the cost of sole ownership. But might there be dangers in communicating about access-based consumption in terms of its affordability, particularly among this segment of consumers? To answer this question, we investigate the effects of framing access-based consumption in terms of two primary benefits: affordability and variety. Results from four studies suggest that although affordability might rationally be of most interest to financially-constrained individuals, framing access-based consumption’s benefits in terms of affordability undermines the happiness they may extract from their consumption relative to framing in terms of variety. This difference emerges because communications focused on affordability re-affirm the negative self-identity financially-constrained individuals perceive as a result of their financial situation. Given these findings, we make clear recommendations for communications related to the access-based economy and this vulnerable set of people.

## Introduction

Many people feel financially constrained, a psychological state that occurs when one’s limited discretionary income fails to match one’s desires (Tully et al., 2015). Such a situation often jeopardizes consumers’ happiness, which in turn can undermine their ability to find success in marriage, friendship, work performance, and health (Lyubomirsky et al., 2005; Hamilton et al., 2019; Hill and Sharma, 2020; Wang et al., 2020). Fortunately, the “sharing economy” has been suggested as a way to overcome this problem, as access-based consumption allows people to access products for a pre-defined time by paying a usage fee (Baumeister et al., 2015), typically more affordable than the costs required to take sole ownership (Lamberton and Rose, 2012).

However, besides resource affordability, research has highlighted that access-based consumption offers another important benefit: access to resource variety (Lawson et al., 2016; Jiang and Tian, 2018). Variety refers to “the number of different items that have been chosen within a purchase history or within an assortment” (Ratner et al., 1999; Ratner and Kahn, 2002; Sevilla et al., 2019). Variety satisfies a fundamental hedonic drive and, if accessed, allows consumers to satisfy dominant social norms (Lähteenmäki and Van Trijp, 1995; Ariely and Levav, 2000; Ratner and Kahn, 2002). Further, access to variety is of particular importance to financially-constrained consumers, for whom variety offers a means of alleviating a low sense of personal control (Yoon and Kim, 2018).

Theoretically, since both saving money and seeking variety are among the motives identified as drivers of access-based service use (Lawson et al., 2016; Smith, 2016; Böcker and Meelen, 2017; Fritze et al., 2021), emphasizing either may be expected to build consumer happiness. However, though prior research has studied the effects of these two types of framing separately (e.g., affordability: Garbinsky et al., 2014; Sharma and Keller, 2017; Snow et al., 2017; Lee-Yoon et al., 2020; variety: Ratner and Kahn, 2002; Chancellor and Lyubomirsky, 2011; Etkin and Mogilner, 2016), little is known about their relative impact, or if this impact differs across levels of consumer financial constraint.

On the one hand, it might be expected that emphasizing affordability as opposed to variety would be the more appealing frame for financially-constrained consumers, as such messaging would directly address this group’s financial insecurity. Indeed, many business communications (e.g., advertisements, blogger posts) promote access-based services that emphasize affordability (see Table 1 for a set of examples). However, we argue that a critical piece may be missing in making this assumption. Our work considers the potential *identity stigma* that financially-constrained consumers may face in this consumption context. Poverty stigma is the worst condition a financially-constrained individual may experience (Hall et al., 2014). In the context of our study, this takes the form of a *rent stigma*, which refers to the association between temporary access (i.e., renting) and the lack of wealth (Kricheli-Katz and Posner, 2020). Thus, affordability-based framing may constitute a “double-edged sword,” as such framing not only can increase one’s sense of financial insecurity but also can strengthen the negative association between one’s undesirable financial condition and their consumption mode (Sharma and Keller, 2017). By contrast, variety-seeking is associated with resource abundance and positive personality signals (Ratner and Kahn, 2002). Further, variety may help consumers dissociate from their current undesirable self (variety is negatively associated with self-continuity; Rifkin and Etkin, 2019). Thus, variety-based framing releases consumers from such a negative stigma – allowing financially-constrained individuals to experience happiness.

**Table 1:** Affordability-based communications in the context of access-based consumption.

Access-based businesses	Communications in terms of affordability	Industry
Haverdash	“Introducing Haverdash: The Most Affordable Fashion Rental Service On The Market For Millennials Looking To Experiment With Style, Color and Trends” (Source: https://www.prnewswire.com/news-releases/introducing-haverdash-the-most-affordable-fashion-rental-service-on-the-market-for-millennials-looking-to-experiment-with-style-color-and-trends-300851482.html)	Clothing and accessories
The black tux	“An Affordable Option for Tux & Suit Rental” (Source: https://www.mysubscriptionaddiction.com/clothing-rental-subscriptions)	
Rocksbox	“Best For Renting Jewelry & Accessories on an Everyday Budget” (Source: https://www.mysubscriptionaddiction.com/clothing-rental-subscriptions)	
Budget rent A car	“Budgeters book here for the best deal.” (Source: https://www.valpons.com/valpons/save-up-to-30-off/)	Transportation
Hertz multi-month	“No money down. No hidden fees. No financing. Multi-Month rentals are worry-free. You’ll get special low multi-month rates on a full range of vehicles, including Hybrids, SUVs and Minivans.” (Source:https://www.hertz.com.au/rentacar/misc/index.jsp?targetPage=multimonth.jsp)	
Zipcar’s stay local plan	“stay local & save: zipcar offers reduced hourly and daily rates in response to covid-19” (Source: https://www.zipcar.com/press/newsroom/stay-local)	
Careem bike	“Bike-sharing allows for easy and affordable access to pedal-assisted bikes!” (Source: https://apps.apple.com/us/app/careem-bike-bike-sharing-app/id1491020032)	
Rebirth fitness	“Rebirth Fitness’s rental program is designed to make your fitness activities easier and more accessible. There is no need to buy the equipment, you can rent quality fitness equipment at an affordable price. Gym Equipment Rentals enable our clients to attain their objectives without the expensive of purchasing equipment or the hassle of dealing with inexperienced suppliers.” (Source: https://www.fitnessplus.com/services/rentals)	Fitness
Breeze ski rentals	“Book your discount ski rentals today and save up to 25%.” “With discounts of up to 25% when you book in advance, plus an extra 20% off for Epic Pass Holders, you’ll get the best value for great gear.” (Source: https://www.skirentals.com/)	Sports
Feather	“Stop spending a fortune upfront. It costs $6,000 on average to furnish a one-bedroom with quality furniture. With Feather, you can rent items starting at $4/mo, and our team handles everything you’d rather not: delivery, assembly, & pickup.” (Source: https://www.livefeather.com/)	Home décor

This research aims to build a comprehensive framework in order to understand the effect of different kinds of benefit framing on financially-constrained consumers’ happiness in the context of access-based consumption. Prior work has discussed how access-based consumption might contribute to financially-constrained consumers’ happiness (Eckhardt and Bardhi, 2016; Bardhi and Eckhardt, 2017). However, little empirical work has been conducted to test how exactly access-based consumption can improve this group’s happiness and with it facilitate a broad range of well-being enhancements (Diener E. D. et al., 1985; Kahneman et al., 1999; Alba and Williams, 2013). Moreover, to our best knowledge, the current research is the first that brings up the marketplace stigmas (Harmeling et al., 2021) that financially-constrained individuals might face in this new consumption context. Therefore, considering both the bright and the dark sides of access-based consumption, our proposed framework delineates the (net) effect of different kinds of framing of access-based consumption on financially-constrained consumers’ happiness and helps businesses and practitioners understand how to better communicate their access-based services’ benefits.

Next, we explicate our theory that communicating the benefits of access-based consumption in terms of resource affordability (vs. variety) can heighten poverty stigma among financially-constrained individuals, reducing the positive effect of access-based consumption on this group’s happiness. We then present four studies that test this proposition, measuring both expected and post-consumption happiness. Finally, we conclude with a general discussion of our findings, theoretical contributions, practical implications, limitations, and future research opportunities.

## Theory and Hypotheses

### Access-Based Consumption and Consumer Well-Being

As an alternative to ownership-based consumption, access-based consumption refers to “transactions that can be market mediated but no transfer of ownership takes place” (Bardhi and Eckhardt, 2012). Scholars have proposed that access-based consumption may enhance consumer well-being in multiple ways (Baumeister et al., 2015; Harding et al., 2019; Fritze et al., 2021). First, and by definition, access-based consumption may improve consumer well-being by allowing consumers to avoid the “burdens” of ownership (Moeller and Wittkowski, 2010), thus reducing their overall stress and anxiety. Beyond this, access-based consumption has been argued to enhance consumer well-being by accelerating consumers’ speed in learning new skills to a greater extent than does ownership (Harding et al., 2019). Access-based consumption’s ability to bolster the well-being of consumers may be particularly crucial under conditions of uncertainty, as it aids them in navigating periods of liminality (i.e., social role transitions; Ozanne and Ozanne, 2020), or as sharing materials helps build collective resilience (Baker and Baker, 2016). Moreover, sharing can bolster well-being by allowing consumers access to a variety of shared goods, such as developmentally appropriate toys for children (Ozanne and Ballantine, 2010), fashion items (Lang, 2018), vehicles (Bardhi and Eckhardt, 2012), bikes (Sun and Ertz, 2021), and furniture (Kapoor and Vij, 2021). More specific to our research, scholars have also proposed that access-based consumption may improve financially-constrained consumers’ well-being, whether by highlighting new job opportunities (Dillahunt and Malone, 2015), or by reducing perceived social inequality (Eckhardt and Bardhi, 2016).

However, scant empirical research has determined whether marketers’ and other communications’ access-based consumption framings may affect such consumers’ ability to extract the hoped-for benefits to happiness. We next argue that, indeed, the way in which access-based consumption is framed for consumers matters. In the following sections, we first delineate variety and affordability as key benefits that access-based consumption can provide to financially-constrained consumers. We then examine how framing access-based consumption in terms of these two benefits can elicit or reduce the negative poverty stigma financially-constrained consumers may face and in turn impact the amount of happiness they experience in access-based consumption.

### Access-Based Consumption’s Potential for Enhancing Financially-Constrained Consumers’ Happiness

The feeling of financial constraint refers to a psychological state in which “people believe their financial situation restricts their desired consumption” (Tully et al., 2015).^1^ Ideally, access-based consumption offers options by which consumers can alleviate the unhappiness that ownership can cause to those who face financial constraints. It may do this in two ways: First, access-based consumption offers access to a wider variety of goods than would sole ownership, where a consumer must select a single item. Financially-constrained individuals often purchase subjectively less desirable options, as their financial constraints require them to trade away desirability for the sake of affordability (Fernbach et al., 2015; Hamilton et al., 2019). Access-based consumption, by offering lower momentary prices, may alleviate the need to make such trade-offs. Second, access-based consumption involves temporary consumption opportunities. Research shows that financially-constrained consumers try to avoid potential future costs (Tully et al., 2015). Since access-based consumption provides temporary use of products at affordable prices, future costs do not need to be incurred when temporary needs are satisfied. Thus, access-based consumption theoretically allows financially-constrained individuals to simultaneously enjoy both low price and choice variety without the “burden” of ownership (Lawson et al., 2016). For instance, a financially-constrained consumer may have little money to buy a vehicle, not to mention to pay for car insurance, parking, and maintenance costs. However, they can easily access a vehicle by paying a small usage fee through access-based services, which does not create the burdens associated with sole ownership. Further, each access-based service use allows the opportunity to drive a different vehicle. Therefore, along the process of repeated access, these consumers can enjoy both lower prices and wider variety than under ownership.

However, despite the aforementioned benefits, the potential stigma that financially-constrained individuals may experience in the context of access-based consumption may minimize the happiness they could derive from those benefits. First, financial constraint *per se* has long been seen as a stigmatizing attribute. As such, it is itself a condition that may lead to negative self-perception (e.g., “financial shame spirals,” Gladstone et al., 2021), which can be escalated to poverty stigma, an extremely negative condition associated with those in poverty (Hall et al., 2014). Besides this potential threat faced by the financially-constrained, research also suggests that access-based consumption may be associated with a specific stigma, such that people believe that temporary access is a default method for those who need to save money. In Western markets, this has been referred to as “rent stigma” (in United States, Cohen et al., 2009; in United Kingdom, Foye et al., 2018; in Israel, Kricheli-Katz and Posner, 2020). For instance, Kricheli-Katz and Posner (2020) show that students are reluctant to temporarily access products even though the benefit of access outweighs that of ownership. Further, home ownership vs. rental has been shown to be a “positional good” for those who lack financial resources (Foye et al., 2018), changing their elevation in the social hierarchy.

It is clear that poverty-related stigma will jeopardize consumer well-being by reducing happiness in one’s life (Mickelson and Williams, 2008). What we do not know then is under what conditions access-based consumption will be more or less likely to heighten poverty stigma among the financially constrained. To answer this question, we next undertake an analysis of the framings in which access-based consumption is presented, specifically in terms of their potential for stigmatization among financially-constrained consumers.

### The Framing of Access-Based Consumption and Poverty Stigma

As mentioned above, resource affordability and variety are two fundamental benefits that access-based consumption can provide to consumers. We argue that framing access-based consumption in terms of affordability may do more harm than good when considering the potential for stigmatization in this context. First, prior research shows that framing an activity in terms of affordability will decrease financially-constrained individuals’ willingness to participate in this activity (Hall et al., 2014; Sharma and Keller, 2017). For instance, in financial planning contexts, financially-constrained individuals are less willing to save money when the task is framed as “saving a penny” vs. “earning a penny” (Sharma and Keller, 2017). Also, they tend to refuse to collect government benefits, such as food stamps and other free benefits, to avoid bolstering their already undesirable self-image (Hall et al., 2014). Additionally, cues that indicate a lack of financial resources will decrease the positive feelings this group of individuals can extract from consumption (Lee-Yoon et al., 2020). For instance, in the gift-giving context, financially-constrained gift-receivers feel less happy about a gift that is framed in terms of saving money vs. saving time (Lee-Yoon et al., 2020).

Consistent with this trend, recent qualitative research by Magnani and Re (2020) shows that although financially-constrained young adults reported a very strong intention to use access-based services, getting temporary access to something that they are not able to own made them more aware of their undesirable financial condition, leading to negative feelings. Thus, framing access-based consumption in terms of affordability may undermine financially-constrained individuals’ self-image by strengthening their sense of being poor, and in turn, more deeply engraining a poverty stigma (Hall et al., 2014).

By contrast, framing access-based consumption in terms of variety may not elicit a sense of poverty stigma in financially-constrained individuals. This is because variety-seeking sends positive signals to social observers (Ariely and Levav, 2000; Ratner and Kahn, 2002), which may help financially-constrained consumers present themselves, rather than simply poor, as fun and interesting individuals. Additionally, consuming variety is associated with resource abundance, as doing so signals that one can afford freedom of choice (Diener et al., 1985; Yoon and Kim, 2018). Therefore, variety-based framing could possibly alleviate, rather than escalate, the sense of poverty stigma among financially-constrained individuals in the context of access-based consumption.

Based on the above arguments, we predict that framing access-based consumption in terms of affordability vs. variety will elicit a heightened level of poverty stigma among financially-constrained individuals. Since poverty stigma is a type of negative identity cue that financially-constrained individuals want to avoid, attaching to a stronger sense of poverty stigma will decrease one’s happiness. Thus, we further predict that due to the heightened level of poverty stigma, financially-constrained individuals will feel less happy when access-based consumption is framed in terms of affordability vs. variety.

We do not expect that the above-proposed relationship exists among those who are financially-unconstrained. First, our theory is built on stigmas in the context of access-based consumption. Since poverty stigma is irrelevant to financially-unconstrained individuals’ identity, and rent stigma may be easily buffered by their desirable financial condition, our proposed effect should have no impact on financially-unconstrained individuals’ self-perception of their financial condition. Second, financially-unconstrained individuals may have achieved the consumption of a variety of products in any type of consumption (i.e., access-based and ownership-based). Thus, the variety framing may not improve these individuals’ view of their financial condition relative to the affordability framing. Therefore, we predict that financially-unconstrained individuals will feel similarly happy in the context of access-based consumption regardless of the framing.

To test our theory, we conducted four studies^2^ in which participants living in the United States imagined accessing or actually accessed products (see Table 2 for a summary of study stimuli). Study 1 tested the interaction effect of financial constraints and framing on happiness in a clothing sharing program. Study 2 replicated the findings in Study 1 using a different financial constraint manipulation in a vehicle sharing context. A replication experiment, Study 3, further tests the proposed framework in a clothing sharing scenario (similar to scenario study 1). Finally, Study 4 tests the proposed mediator in a pen-sharing context where college students were invited to use shared pens in the laboratory and then indicated their post-consumption happiness with their experience. Study 4 also explored the role of psychological ownership in alleviating the poverty stigma felt by financially-constrained individuals under different access-based consumption framings (see Table 3 for a summary of results across four studies).

**Table 2:** Study 1, 2, and 4 stimuli summary.

**Study 1. Clothing consumption**		
Financial constraints	**Salient** condition (Tully et al., 2015): “Everyone has financial constraints in their lives, but the factors that contribute to these constraints tend to vary. What are the factors that require you to be careful with how you spend your money? What limits your monthly discretionary income? Include the aspects of your current situation that most contribute to your financial constraints (e.g., mortgage or rent, family expenses, uncertainty of future income, health care costs, student loans, lack of income, limited savings, bills that need to be paid, expensiveness of entertainment …). Please be as detailed as possible, and write at least a couple of sentences.”	
Framing	**Affordability** condition: “…… Finally, you decide that you should make the choice that you can best afford right now. You think the best thing you can afford to do is to spend $1,000 to rent branded business professional clothes from an online retailer. You do this because you know this is the most you can afford.” **Variety** condition: “…… Finally, you decide that you should make the choice that will give you the most variety and choice possible. You think you can get the most variety by spending about $1,000 to rent branded business professional clothes from an online retailer. You do this because you know this option will provide you with a great deal of variety.” **Purchase** condition: “…… Finally, you decide that you should purchase clothing for the next year. You think the best move is to spend $1,000 to buy some branded business professional clothes from an online retailer.”	
**Study 2. Vehicle accessing**		
Financial constraints	**Constrained** condition: “Suppose that you will start a new job in a new city within a month. The compensation is only enough to cover your basic cost-of-living. At the end of this job, you might not have savings after spending the money from the compensation.” **Unconstrained** condition: “Suppose that you will start a new job in a new city within a month. The compensation is abundant enough to cover more than your basic cost-of-living. At the end of this job, you will have a large amount savings after spending the money from the compensation.”	
Framing	**Affordability** condition: “The vehicle sharing program is called ‘Afford2Ride.’ It aims to provide every user with affordable car-riding experiences……. You’ve decided to opt into this vehicle sharing program when you start your job in that city in order to enjoy various car-riding experiences.” **Variety** condition: “The vehicle sharing program is called ‘Variety2Ride.’ It aims to provide every user with a variety of car-riding experiences…… You’ve decided to opt into this vehicle sharing program when you start your job in that city in order to spend the least amount of money on transportation.”	
**Study 4. Paint Pen Laboratory Study**		
Financial constraints	Similar to study 1’s procedures	
Framing	**Affordability** condition	**Variety** condition
**Access** mode: “Using SHARED paint pens means that you are one of the participants who can borrow these SHARED pens for a short-period of time.”	We provide this sharing program because we know people cannot usually afford to own watercolor paint equipment. You get access these shared watercolor paints pens affordably, thanks to Afford2Paint.	We provide this sharing program because we know people do not usually have access to a large variety of types of watercolor paint equipment. You get access to a wide variety of watercolor paint pens, thanks to Variety2Paint.
**Psychological ownership** mode: “Having YOUR OWN paint pens means that you are the only person who can use YOUR pens for a long period of time.”	We provide this vendor because we know people cannot usually afford to own watercolor paint equipment. You have your own affordable watercolor paint pens, thanks to Afford2Paint.	We provide this vendor because we know people do not usually own a large variety of types of watercolor paint equipment. You have your own wide variety of watercolor paint pens, thanks to Variety2Paint.

**Table 3:** Studies 1–4 results summary.

Studies	Financial constraints	Framing	Mean	Std. dev.	Cell size	Focal pairwise comparisons (Bonferroni-adjusted tests)
**Study 1** clothing (DV: happiness)	Salient	Affordability	3.48	1.77	52	Affordability vs. variety: *p* =0.02 Purchase vs. variety: *p* =0.71
		Variety	4.32	1.76	68	
		Purchase	4.65	1.35	68	
	Control	Affordability	4.19	1.73	65	Affordability vs. variety: *p* =0.91 Purchase vs. variety: *p* =0.002
		Variety	3.87	1.79	46	
		Purchase	5.02	1.26	47	
**Study 2** vehicle (DV: happiness)	Constrained	Affordability	3.07	1.13	60	Affordability vs. variety: *p* =0.01
		Variety	3.51	0.98	76	
	Unconstrained	Affordability	3.63	0.91	57	Affordability vs. variety: *p* =0.66
		Variety	3.55	0.92	52	
**Study 3** clothing (DV: happiness)	Constrained	Affordability	2.88	1.63	100	Affordability vs. variety: *p* =0.07 Control vs. variety: *p* =0.005 Purchase vs. variety: *p* >0.99
		Variety	3.49	1.53	83	
		Control	2.69	1.64	108	
		Purchase	3.42	1.64	109	
	Unconstrained	Affordability	3.33	1.74	102	Affordability vs. variety: *p* =0.10 Control vs. variety: *p* >0.99 Purchase vs. variety: *p* <0.001
		Variety	3.87	1.64	116	
		Control	3.68	1.78	94	
		Purchase	5.07	1.52	94	
**Study 4** paint pens (DV: happiness)	Salient	Affordability	3.10	1.18	115	Affordability vs. variety: *p* =0.008
		Variety	3.49	1.08	106	
	Control	Affordability	3.18	1.16	105	Affordability vs. variety: *p* =0.90
		Variety	3.20	1.09	116	
(Mediator: poverty stigma)	Salient	Affordability	3.60	0.83	115	Affordability vs. variety: *p* =0.03
		Variety	3.36	1.03	106	
	Control	Affordability	3.37	0.91	105	Affordability vs. variety: *p* =0.39
		Variety	3.46	0.86	116	

## Study 1

### Method

#### Participants and Design

Study 1 employed a 2 (financial constraints: salient vs. control) × 3 (framing: affordability vs. variety vs. purchase) between-subjects design. Based on the roughly 10–20% rate of problematic data on MTurk (Kees et al., 2017; Buhrmester et al., 2018; Chmielewski and Kucker, 2020), we planned to recruit 370 participants from Amazon’s Mechanical Turk (MTurk) to allow each cell to have about 50 usable participant responses per cell. Our final sample contained 346 participants (194 women, 148 men, four missing information; *M*_age_=41.52; *M*_income_=$59,853; exclusions explained below).

#### Procedure

In this study, participants first completed the manipulation of the financial condition (or a control task) and then evaluated an option for them to get clothes. Besides the two framing conditions in which participants access clothes, we include an ownership condition in which participants spend the same amount of time to get their clothes for the same length of period. Though the comparison between ownership and access is not our focus in this paper, adding this condition allows us to gauge how much happiness financially-constrained consumers may gain or lose through accessing vs. owning products, holding total cost constant.

After reading an introductory page, participants were randomly assigned to either the financial-constraints salient condition or the control condition. In the constraint salient (*control*) condition, participants were asked to write down their sources of financial constraints in at least two sentences (*write down 10 facts they knew that were true*; Tully et al., 2015), followed by a manipulation check question: participants rated the extent to which they felt financially constrained on a seven-point scale (1=“Not at all”; 7=“Very much”). Based on information from the previous work (Sharma and Keller, 2017; Yoon and Kim, 2018), we expected that this manipulation would be subtle (Tully et al., 2015) and that the framing conditions might impact this manipulation. Thus, we moved the manipulation check above the dependent measure so as to measure the distinct effect of the manipulation (Perdue and Summers, 1986). Following the manipulation check, participants completed a filler task (i.e., type the letters in a picture). Participants who did not follow instructions when completing the financial constraints salient manipulation (*N*=9) or the control task (*N*=8), and those who did not type the letters correctly in the filler task (*N*=12) were excluded from data analyses (*N*=24 with five failing both).

After completing the filler task, participants imagined the following scenario: “Suppose that you have just finished a new degree and will start a new job in a new city in 1week. Due to the nature of your new job, your dress code for work is business professional from Monday to Thursday. Suppose that you do not have enough of the right clothing, shoes, and accessories to wear. That is, you just do not have much that falls into the ‘business professional’ category. Now you start to think about ways to get your clothes for work.” Then, participants were randomly told that they could spend $1,000 on a year-long clothing supply through one of the three options: (1) join a clothing sharing program as it is “the best option you can afford” (affordability framing), (2) join a clothing sharing program as “it is the most varied option” (variety framing), or (3) buy clothing from an online retailer. Note that in all cases, participants imagined engaging in a purchase through an online retailer in order to avoid the creation of channel-related confounds.

Following this, participants reported how happy/satisfied they would feel if they got their clothing in the way described to them (variety-based access v. affordability-based access v. ownership) on a seven-point scale (1=“Not happy/satisfied at all”; 7=“Very happy/satisfied”; happiness index, *ρ*=0.87, Etkin and Mogilner, 2016). For exploration purposes, we also measured participants’ sense of fulfillment (1=“Not fulfilled at all”; 7=“Very fulfilled”), another component of subjective well-being driven by the perceived meaningfulness of a task and achieved by satisfying higher-level needs (Diener and Lucas, 2000); additional emotions commonly measured in the consumer financial constraint literature (i.e., pride, shame, embarrassment; scale: 1=“Not at all”; 5=“Very”; see a brief summary: Goldsmith et al., 2020); and participants’ word-of-mouth tendency related to their acquisition of clothing [item 1: “I would want to talk with them about it”; item 2: “I would want nobody to know it” (reverse-code); scale: 1=“Not at all”; 5=“Very”]. After that, participants were given a chance to choose between renting and purchasing clothes to allow us to explore if framing impacts financially-constrained participants’ intention to use access-based services.

Besides the above measures, participants responded to a series of other questions for exploratory purposes [e.g., their perception of the variety-seeking behavior (e.g.*, “It is inappropriate for people to prioritize variety in their daily consumption”*), annual clothing shopping budget, the realism of the scenario, their clothing sharing history (*all of those participating had rented clothing before*); see online Appendix for details] and answered demographic questions.

### Results and Discussion

We estimated all models both with and without covariates (i.e., annual expenditure on clothing and perceived realism of the scenario, age, gender, and income). No reported significant results below become non-significant when covariates are included in the model. Therefore, all subsequent results are reported without the inclusion of any covariates. Additionally, no reported effects below substantively change when using the full sample, without exclusions (i.e., *N*=370). Details of effects of covariates^3^ and robustness checks using the full sample are included in the Web Appendix.

#### Manipulation Check

A two-way ANOVA with financial constraints, framing, and their interaction term as independent variables and the feeling of financial constraint as the dependent measure revealed only a significant main effect of financial constraints [*F*(1, 340)=29.98, *p*<0.001]. As expected, participants in the constraints salient condition (*M*=5.43, SD=1.36) felt more constrained than their counterparts in the control condition (*M*=4.54, SD=1.71).

#### Perceived Happiness

**Figure 1:**
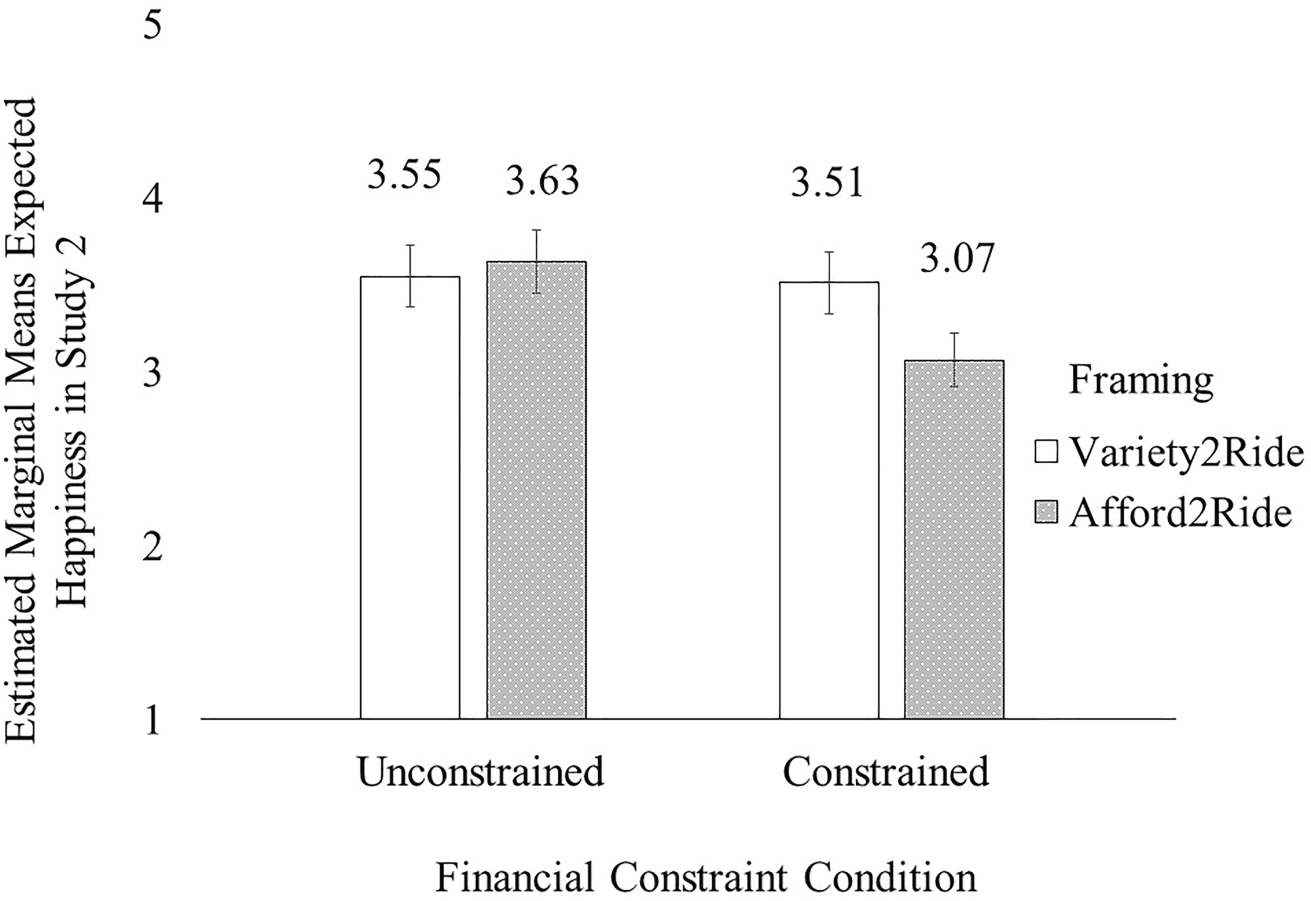
The financial constraints × framing interaction effect on happiness in Study 1.

A two-way ANOVA with financial constraints, framing, and their interaction term as independent variables and happiness as the dependent variable revealed a significant main effect of framing [*F*(2, 340)=11.45, *p*<0.001] qualified by a significant two-way interaction [*F*(2, 340)=3.76, *p*=0.02; see Figure 1].

Overall, participants who purchased clothes (*M*_purchase_=4.80, SD=1.32) expected to feel happier than those who accessed clothing, regardless of whether access was framed in terms of variety-seeking (*M*_variety_=4.14, SD=1.78, *p*=0.002) or affordability (*M*_affordability_=3.88, SD=1.77, *p*<0.001; affordability vs. variety: *p*=0.71). Further, participants in the control condition expected to feel similarly happy when they accessed to save money (*M*_control+affordability_=4.19, SD=1.73) and to seek variety (*M*_control+variety_=3.87, SD=1.79; *p*=0.91). However, pairwise comparisons showed that as expected, participants in the salient financial constraint condition expected to feel less happy when accessing clothes to save money (*M*_salient+affordability_=3.48, SD=1.77) than when seeking variety (*M*_salient+variety_=4.32, SD=1.76; *p*=0.02);

Further, we also found that, in the control condition, purchasing led to higher expected happiness (*M*_control+purchase_=5.02, SD=1.26) than accessing for variety (*p*=0.002); however, in the financial constraint salient condition, variety-seeking and purchase made participants similarly happy (*M*_salient+purchase_=4.65, SD=1.35; *p*=0.71).

#### Other Measures

Several similar ANOVAs were conducted with each of the following as the dependent measures: fulfillment, pride, shame, embarrassment, word-of-mouth tendency, and judgment of variety-seeking. None of these revealed a significant constraint × framing interaction effect, except for the word-of-mouth tendency. Although the interaction effect on word-of-mouth tendency was significant [*F*(2, 340)=3.21, *p*=0.04], our focal pairwise comparison was non-significant. Participants had a similar word-of-mouth tendency when they accessed clothes to save money and seek variety (*M*_salient+affordability_=2.32, SD=1.16; *M*_salient+variety_=2.50, SD=0.98; *p*>0.99). The only significant pairwise comparisons were between ownership condition and the other two framing conditions, such that when participants’ financial constraints were made salient, they had a higher word-of-mouth tendency in the purchasing condition (*M*_salient+purchase_=3.37, SD=0.90) than the affordability condition and the variety condition, respectively (*ps*<0.001). All of these suggest that our framework is specific to the happiness that can be obtained from access-based consumption. We also conducted a binary logistic regression analysis with the binary choice (i.e., purchase=1, rent=0) as the dependent variable, and financial constraints, framing, and their interaction term as the independent variables to test whether there was an effect on participants’ intention to access clothes (*N*_rent_=31, *N*_purchase_=315). We did not find a significant effect (see online Appendix).

#### Discussion

Study 1 provides preliminary evidence that consumers do prefer owning (vs. accessing) products, holding all else equal. However, in the context of access-based consumption, happiness depended both on their financial constraints and the framing of the access-based opportunity. Consistent with our theory, financially-constrained consumers who think about access-based consumption in terms of affordability are significantly less happy than those who think about it in terms of variety-seeking. In fact, framing access-based consumption in terms of variety-seeking can offer financially-constrained consumers happiness equivalent to ownership – thus delivering on access-based consumption’s promise.

However, this study also highlights the specificity of these effects both to happiness and to consumers for whom financial constraint is salient. For consumers who had not had their financial constraints brought to mind, ownership *always* created more happiness than access, regardless of framing. Further, we see no changes in other emotions or experiences driven by framing. Thus, it appears that firms that want to build happiness, specifically, may benefit from framing access-based consumption in terms of variety, particularly for financially-constrained consumers.

## Study 2

### Method

#### Participants and Design

In Study 2, 305 participants from Amazon’s Mechanical Turk (MTurk) were randomly assigned to a 2 (financial situation: constrained vs. unconstrained) × 2 (framing: variety vs. affordability) between-subjects design. Sixty participants did not follow instructions when responding to one or more open-ended questions and were excluded from data analyses (see below for details). Therefore, the final dataset contained 245 respondents (122 women, 123 men; *M*_age_=37.48; *M*_income_=$58,660).

#### Procedure

In this study, we manipulated the salience of financial constraints by leveraging participants’ job compensation and expenditure in a fictitious scenario. Specifically, all participants imagined starting a new job in a new city within a month. Then, participants in the financially constrained (*unconstrained*) condition read: “The compensation is only enough (*abundant*) to cover your basic cost-of-living (*more than your basic cost-of-living*). At the end of this job, you might not have (*will have a large amount of*) savings after spending the money from the compensation.” To strengthen this manipulation, participants then completed an elaboration task by filling in the blanks in four sentences: “(1) If I were to take this job, I would feel financially ______; (2) During my work in this city, I would feel financially _______; (3) After I bought something using the compensation, I would feel financially _______; (4) The amount of compensation would make me feel financially ______.” Participants (*N*=54) who provided inconsistent responses (e.g., “loss/drop/better/gain” and “insecure/secure/stable/stable”), responses contrary to the assigned condition (e.g., “constrained/restricted/scared/annoyed” in the financially unconstrained condition), or irrelevant responses (e.g., “scals/saint louis/good/50” and “not now/yes/no feel/10000”) were excluded from data analyses to ensure data quality (Kennedy et al., 2020).

Following this elaboration task, participants were told that they were looking for a way to get a car to commute and that the company that offered the job partnered with a vehicle sharing program to help employees get access to vehicles with a 15% below-market-rate discount. Participants were then randomly introduced to either the “Afford2Ride” vehicle sharing program, which aimed to provide every user with affordable car-driving experiences (affordability framing), or the “Variety2Ride” program, which aimed to provide every user with a variety of car-driving experiences (variety framing). After participants moved to the next page, they were asked to type down the name (provided again) of the vehicle sharing program in an open-ended box as an instructional manipulation check; four participants failed and were excluded (Oppenheimer et al., 2009).

Next, participants indicated their daily willingness-to-pay (WTP) by moving a slider from $0 to $30. They were also asked to briefly explain their WTP in an open-ended box; 30 participants failed at this point to provide a relevant response (e.g., “nice,” “good survey,” “jyhguyt8”) and were excluded.

After the WTP measure, participants indicated how happy/satisfied they would feel if they used this vehicle sharing program (1=Not at all; 5=Very; happiness index, *ρ*=0.80). Next, similar to Study 1, they also responded to a set of exploratory variables (e.g., emotions and word-of-mouth tendency), and we would not discuss them further.

Finally, participants completed a manipulation check for the feeling of financial constraints (i.e., “How financially constrained would you feel if you were the person in this scenario”; scale: 1=“Not at all” and 5=“Very”) and indicated the perceived realism of the scenario, their driving history, age, gender, and income.

### Results and Discussion

We estimated all models both with and without covariates (i.e., car ownership, driver’s license condition, and perceived realism of the scenario, gender, age, and income). Again, no reported significant results below become non-significant when covariates are included in the model. Therefore, all subsequent results are reported without the inclusion of these measures. Also, no reported effects below substantively change when using the full sample before data exclusion. Details of effects of covariates^4^ and robustness checks using the full sample are included in the Web Appendix.

#### Manipulation Check

A 2 (financial constraints: constrained vs. unconstrained) × 2 (framing: variety vs. affordability) ANOVA with the feeling of financial constraint as the dependent measure revealed only a significant main effect of financial constraints [*F*(1, 241)=212.52, *p*<0.001]. The constraints × framing interaction term was non-significant [*F*(1, 241)=0.21, *p*=0.64]. As expected, participants in the constrained condition (*M*_constrained_=4.21, SD=0.93) felt more financially-constrained than those in the unconstrained condition (*M*_unconstrained_=2.35, SD=1.05).

#### Daily Willingness-to-Pay

A two-way ANOVA with financial constraints, framing, and their interaction term as independent variables and WTP as the dependent variable revealed no main effects, nor a significant interaction effect, suggesting that framing did not impact participants’ monetary valuation of access-based consumption.

#### Perceived Happiness

**Figure 2:**
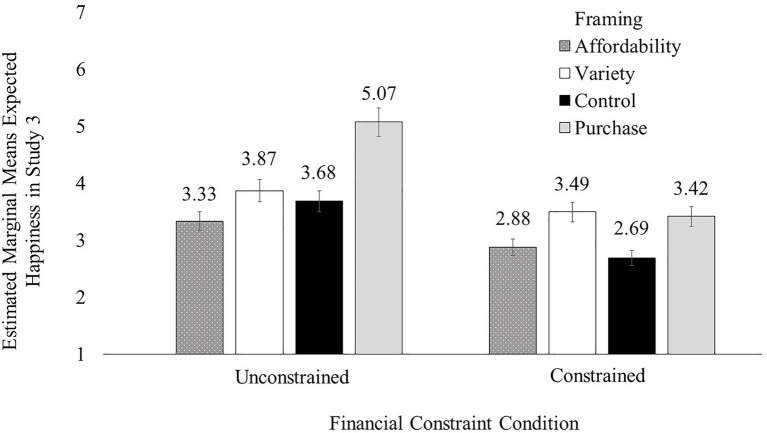
The financial constraints × framing interaction effect on happiness in Study 2.

A similar two-way ANOVA with happiness as the dependent variable revealed a significant main effect of financial constraints [*F*(1, 241)=5.50, *p*=0.02] followed by a significant two-way interaction [*F*(1, 241)=4.30, *p*=0.04; see Figure 2]. As expected, participants in the financially-constrained condition expected to feel less happy when accessing vehicles to save money (*M*_constrained+affordability_=3.07, SD=1.13) than to seek variety (*M*_constrained+variety_=3.51, SD=0.98; *p*=0.01); this difference was non-significant among those in the unconstrained condition (*M*_unconstrained+affordability_=3.63, SD=0.91; *M*_unconstrained+variety_=3.55, SD=0.92; *p*=0.66).

#### Other Measures

Similar ANOVAs were conducted with each of the following as the dependent measure: pride, shame, embarrassment, and word-of-mouth tendency. Again, similar to Study 1, none of these revealed a significant constraint × framing interaction effect, suggesting that our framework is specifically about happiness participants can extract from consumption.

#### Discussion

The results in Study 2 replicated Study 1’s findings, showing their generalizability to variant manipulations of financial constraint salience and other product categories where access-based consumption is common. This replication in Study 2 also suggests that our proposed framework works across the different lengths of access-based consumption (i.e., yearly clothing sharing in Study 1 and daily vehicle sharing in Study 2). In addition, age, gender, and income did not predict happiness in both studies, showing that the robustness of these effects is above and beyond demographics.

## Study 3

Study 3 has three objectives. First, neither Study 1 nor Study 2 had a control framing condition in which no benefit of access-based consumption was emphasized. Without such a control framing, it is impossible to verify whether it is the variety framing that increases happiness or the affordability framing that decreases happiness. Therefore, we added a control framing in Study 3 to better address the limitations in Studies 1 and 2. Second, the variety framing in Study 1 (i.e., “the most varied option”) might be confusing. Study 3 aims to address this issue by using a clearer variety framing in the same clothing consumption context as in Study 1. Third, though results in the prior two studies are robust to the inclusion or exclusion of participants who failed attention checks, further replication is warranted to show the effect’s reliability. Importantly, since Study 3 was launched during the COVID-19 pandemic, it also tests whether our proposed effects hold with the impact of the pandemic (Hossain, 2021). To sum up, we offer this replication, along with the addition of a control condition and a clearer variety framing, in Study 3.

### Method

#### Participants and Design

In Study 3, 806 participants from Amazon’s Mechanical Turk (MTurk) were randomly assigned to a 2 (financial situation: constrained vs. unconstrained) × 4 (framing: variety, affordability, control, purchase) between-subjects design (416 women, 386 men, four non-binary gender; *M*_age_=40.67; M_income_=$72,617).

#### Procedure

All participants imagined starting a new job in a new city within a month, where their dress code was business professional from Monday to Thursday. Then, they were randomly assigned to one of the financial situation conditions. In the financially constrained condition, participants read, “The compensation is only enough to cover your basic cost-of-living. At the end of this job, you might have nothing left after spending the money from your compensation.” In the financially unconstrained condition, participants read: “The compensation is abundant enough to cover more than your basic cost-of-living. At the end of this job, you will have a large amount of leftover after spending the money from your compensation.”

Next, as in Study 2, all participants completed an elaboration task. Specifically, they were asked to indicate how they would feel if they were in this situation by filling in the blanks in three sentences: “(1) If I were to take this job, I would feel financially ______; (2) During my work in this city, I would feel financially _______; (3) The amount of compensation would make me feel financially ______.”

Following this, participants were told: “Due to the nature of your new job, your dress code for work is business professional from Monday to Thursday. Suppose that you do not have enough of the right clothing, shoes, and accessories to wear. That is, you just do not have much that falls into the ‘business professional’ category. Now, you start to think about ways to get your clothes for work.” Then, participants were randomly assigned to one of the framing conditions. Participants in the variety condition were told: “Finally, you decide that you should make the choice that you can get the largest variety of clothing for the next year. You think you can get the most variety by spending about $1,000 to rent branded business professional clothes from an online clothing sharing company. You do this because you know this option will provide you with a great deal of variety.” Participants in the affordability condition were told: “Finally, you decide that you should make the choice that you can best afford to get your clothing for the next year. You think the best thing you can afford to do is to spend $1,000 to rent branded business professional clothes from an online clothing sharing company. You do this because you know this is the most you can afford.” Participants in the control condition were told: “Finally, you decide that you should rent clothing for the next year. You think the best move is to spend $1,000 to rent some branded business professional clothes from an online clothing sharing company.” Participants in the purchase condition were told: “Finally, you decide that you should purchase clothing for the next year. You think the best move is to spend $1,000 to buy some branded business professional clothes from an online retailer.”

After being informed of how they would get their work wardrobe, participants indicated their expected happiness by responding to “How happy/satisfied would you feel about the choice you made about your clothing?” (scale: 1=“Not happy/satisfied at all”; 7=“Very happy/satisfied”; *ρ*=0.90).

Next, we also measured the perceived product quality (“Do you think the quality of the clothes you get this way is ___?” on the scale: 1=“Extremely low”; 7=“Extremely high”). We also explored whether framing might change participants’ decision of renting clothing using the same dichotomous measure: “If you had the choice, would you prefer to obtain your clothes in this scenario by renting or purchasing?”

Finally, participants responded to the manipulation check (1=“Not at all”; 4=“Moderately financially constrained”; 7=“Extremely financially constrained”) and a few other measures (i.e., whether rented clothing before, annual budget on clothing shopping, the perceived realism of the scenario, and demographic information; given that these measures were not relevant in prior studies, they were not used in the analysis and will not be discussed further).

### Results and Discussion

#### Manipulation Check

A 2×4 ANOVA with the feeling of financial constraint as the dependent measure revealed a significant main effect of financial constraint condition [*F*(1, 798)=1669.10, *p*<0.001] and a significant main effect of framing [*F*(3, 798)=12.85, *p*<0.001], qualified by a significant two-way interaction [*F*(3, 798)=5.69, *p*<0.001]. In general, those in the constrained condition (*M*_constrained_=6.30, SD=0.95) felt more financially constrained than those in the unconstrained condition (*M*_unconstrained_=2.59, SD=1.59; *p*<0.001). Replicating the manipulation check results in Study 2, we found that pairwise comparisons between each two types of framing were non-significant when participants felt financially constrained (*M*_constrained+affordability_=6.42, SD=0.81; *M*_constrained+variety_=6.01, SD=1.02; *M*_constrained+control_=6.47, SD=0.95; *M*_constrained+purchase_=6.23, SD=0.98; *ps*>0.10). However, unexpectedly, participants in the unconstrained condition felt more financially constrained when they accessed to save money (*M*_unconstrained+affordability_=3.33, SD=1.84) than in each of the other conditions (*M*_unconstrained+variety_=2.33, SD=1.40; *M*_unconstrained+control_=2.50, SD=1.63; *M*_unconstrained+purchase_=2.21, SD=1.21; *ps*<0.001). This suggests that the affordability framing in the context of access-based consumption might make even unconstrained people feel chronically financially constrained. As movement in the unconstrained condition is not focal to our hypotheses, we proceed with our analysis following the same design, including both factors in the model.

#### Perceived Happiness

**Figure 3:**
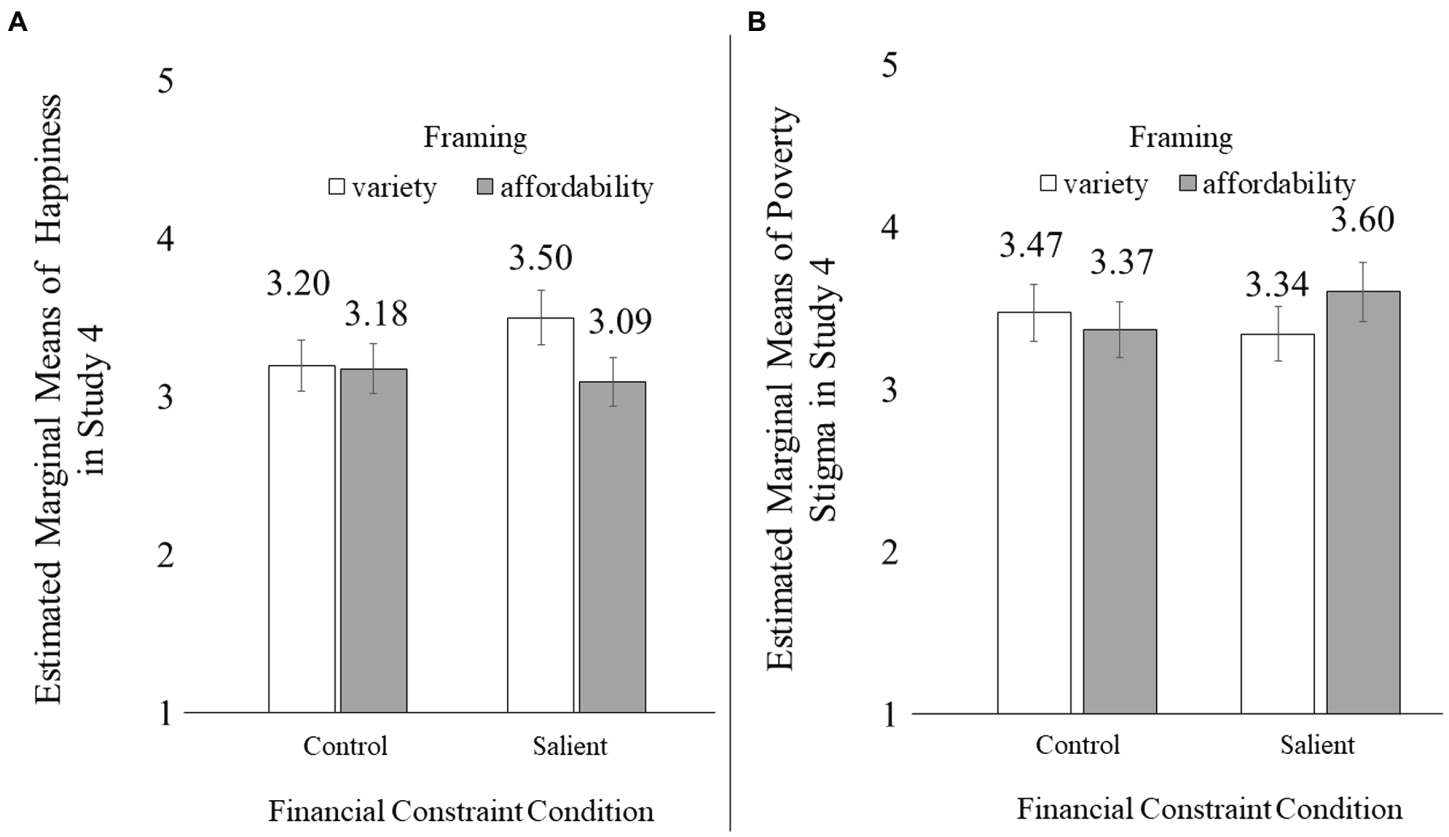
The financial constraints × framing interaction effect on happiness in Study 3.

A similar 2×4 ANOVA with happiness as the dependent variable revealed a significant main effect of financial constraints [*F*(1, 798)=55.79, *p*<0.001] and a significant main effect of framing [*F*(3, 798)=20.67, *p*<0.001], qualified by a significant two-way interaction [*F*(3, 798)=6.44, *p*<0.001; see Figure 3].

As expected, participants in the constrained condition expected to feel happier when accessing in order to gain variety (*M*_constrained+variety_=3.49, SD=1.53) than to save money (*M*_constrained+affordability_=2.88, SD=1.63; *p*=0.07) or when no reason was given for access-based consumption (*M*_constrained+control_=2.69, SD=1.64; *p*=0.005). Those differences were non-significant among those in the unconstrained condition (*M*_constrained+variety_=3.87, SD=1.64; *M*_constrained+affordability_=3.33, SD=1.74; variety vs. affordability: *p*=0.10; *M*_constrained+control_=3.68, SD=1.78; variety vs. control: *p*>0.99). The difference between affordability framing and control framing was non-significant (*ps*>0.88) regardless of whether participants had salient financial constraints or not.

#### Perceived Quality

We then conducted a similar 2×4 ANOVA with perceived quality as the dependent measure. The results did not reveal any significant effects (*ps*>0.10), allowing us to rule out perceived quality as a potential mediator.

#### Exchange Mode Preference

We then conducted a binary logistic regression analysis with participant choice (purchase=1, rent=0; *N*_*r*ent_=102) as the dependent variable to test whether financial constraints and framing would jointly impact people’s preference for exchange mode. The results did not reveal any significant effects (*ps*>0.25).

#### Discussion

Study 3 provides evidence of our findings’ robustness. We note that we find results that are generally consistent with prior work, despite data collection during a global pandemic. Financially-unconstrained consumers are happier owning than accessing, as indicated by the disproportionately high happiness expressed among unconstrained purchasers. Simply, owning under conditions where doing so creates no strain makes us quite happy – even in a world where access-based consumption is sometimes argued to change peoples’ attitudes toward ownership as a whole (e.g., Morewedge et al., 2020). By contrast, ownership does not create the same effect among financially constrained consumers.

The addition of an unframed control condition is also instructive: In the absence of any framing (in the control condition), it appears that financially unconstrained consumers are made approximately as happy as those who encounter variety-focused framing. By contrast, the happiness that financially-constrained consumers derive from an unframed access-based consumption situation was more like that derived from one framed in terms of affordability. In the absence of other information, these more constrained consumers may naturally assume that access is only chosen because it offers their only path to consumption.

More important for the current investigation, as before, we find that access-based consumption framed in terms of affordability creates less happiness than that framed in terms of variety for consumers facing financial constraints. We designed Study 4 to further test our theory in light of experienced consumption, capturing a measure of poverty stigma as our proposed mediator.

## Study 4

In contrast to the previous studies, in which participants imagined accessing shared products and indicated their expected happiness, participants in Study 4 accessed shared products in the laboratory and then indicated their post-consumption happiness.

As an exploratory effort, we also manipulate psychological ownership in the next study. Recent work has suggested that given recent changes in the market – specifically those driven by the rise of more “liquid” forms of consumption – different levels of psychological ownership may offer firms a means of altering consumers’ experience (Morewedge et al., 2020). Psychological ownership is defined as “the state in which individuals feel as though the target of ownership or a piece of that target is ‘theirs’” (Pierce et al., 2001). The previous research shows that psychological ownership has a similar effect to that of ownership on product evaluation and consumer-product identification (Reb and Connolly, 2007; Peck and Shu, 2009; Kirk et al., 2017). Recent literature demonstrates that strengthening people’s psychological ownership of shared goods can increase their willingness to access vs. own products (Bagga et al., 2019; Fritze et al., 2020).

Given these past findings, we manipulate psychological ownership in this experiment for two reasons. First, it may be that in the prior studies, financially-constrained consumers’ happiness is shaped by frustration: not only are they still paying for access, they still do not *feel* like they own the good. This may, in itself, undermine happiness. Second, higher psychological ownership might mitigate our effect. If consumers feel, subjectively, that an accessed good is “theirs,” they may feel less subject to the rent stigma. Therefore, psychological ownership might either have a main effect on happiness from access-based consumption or alleviate the stigma associated with temporary access. However, it is unknown whether psychological ownership of shared goods can reduce the poverty stigma felt by financially constrained individuals to the point that affordability-based framing no longer undermines their happiness. We test this possibility in this study.

### Method

#### Participants and Design

Four hundred and forty-one undergraduate students from two northeastern universities in the United States completed this study in exchange for extra course credit (234 students in university A) or for a $10 compensation (207 students in university B; school coding: A as “1” and B as “2”). Participants were randomly assigned to a 2 (financial constraints: salient vs. control) × 2 (framing: variety vs. affordability) × 2 (psychological ownership: high vs. low) between-subjects design (282 women, 159 men; *M*_age_=22.17).

#### Procedure

After reading an introductory page, participants first completed demographic questions and then were randomly assigned to either the financial constraints salient condition or the control condition, following the same procedure as that used in Study 1 (Tully et al., 2015).

Next, in an ostensibly unrelated “painting activity,” participants first indicated if they had high-quality watercolor paint pens with them. They then were told that the laboratory had partnered with a company that would provide such equipment for participants to complete this study.

Then, participants were randomly assigned to either a high psychological ownership condition or a low psychological ownership condition. We designed this manipulation to be consistent work showing that psychological ownership can be primed, elicited by imagination (Kamleitner, 2015), or mere touch (Peck and Shu, 2009), without requiring changes in post-access retention or other consequences. Specifically, participants in the *high psychological ownership* condition read: “For this task, the lab would like to *give you* some premium ARTEZA watercolor paint equipment. You will *keep your own* watercolor paint equipment for *a long period of time* in this study session. These pens *are yours* because of your *hard-working participation* in the lab. *Having your own paint pens* means that *you are the only person who can use your pens for a long period of time*,” and participants in the *low psychological ownership* condition read: “For this task, our lab would like to provide you an opportunity to *borrow some* premium ARTEZA watercolor paint equipment. You will be able to *borrow the shared* watercolor paint equipment for *the short period of time* in this study session. These paint pens *are shared with you* because of *your attendance* in the lab. Using *shared paint pens* means that *you are one of the participants who can borrow these shared pens for a short period of time*.” In both cases, participants were told that they would return the pens prior to leaving the laboratory.

Following this, participants were randomly assigned to the variety framing condition (i.e., using paint pens from a “vendor” called “Variety2Paint” in the high psychological ownership condition, or a “pen sharing” program called “Variety2Paint” in the low psychological ownership condition) or the affordability framing condition (i.e., using paint pens from a vendor called “Afford2Paint” in the high psychological ownership condition, or a pen sharing program called “Afford2Paint” in the low psychological ownership condition).

Participants then picked up a bag of four-color premium ARTEZA watercolor paint pens from a basket located in the front end of the laboratory room. Since naming (Stoner et al., 2018) and marking (Kirk et al., 2017) are typical procedures to induce psychological ownership, participants were required to write down their nicknames (*write down the word “Shared”*) on the white label of the bag “because they own (*share*) it.”

After the above procedures, all participants painted for up to 5min on a piece of premium paper for watercolor painting (size: 6″×9″). A 5-min timer was presented on the computer screen in the laboratory. After 5min, participants were asked to stop painting and responded to questions related to their painting activity.

We first asked about participants’ general liking of this painting activity (1=“Not at all,” 5=“Very”). Then, participants responded to the happiness measure (i.e., happy and satisfied; *ρ*=0.76) and a few other items (i.e., smart, intelligent, interesting, and bored) on the same screen (1=“Not at all”; 5=“Very”; the order of items was randomized).

Following the dependent measure, participants indicated their sense of poverty stigma, adapted based on participants’ condition. Specifically, participants in the Variety2Paint (*Afford2Paint*) low psychological ownership condition were asked: “Accessing shared watercolor paint equipment from Variety2Paint (*Afford2Paint*) makes me feel like a/an _______ person,” and participants in the Variety2Paint (*Afford2Paint*) high psychological ownership condition were asked: “Having my own watercolor paint equipment from Variety2Paint (*Afford2Paint*) makes me feel like a/an _______ person.” They all responded to this question on a seven-point bipolar scale anchored by five pairs of stigmatized poverty-related traits used in prior research (Lindqvist et al., 2017)^5^: unreliable/reliable, irresponsible/responsible, incompetent/competent, poor/wealthy, and lazy/hard-working. Factor analysis showed that these five reverse-coded items loaded onto one factor (Eigen value=3.19, the perception of variance explained: 63.84%). Thus, we combined them to form a poverty stigma index (*α*=0.85), which served as our proposed mediator.

Finally, participants answered a manipulation check of psychological ownership: “In this painting activity, to what extent did you feel that you owned these watercolor paint pens yourself?” (scale: 1=“Not at all”; 7=“To a great extent”). We also asked if they encountered any difficulty in using the paint pens.

### Results and Discussion

We estimated all models both with and without covariates [i.e., gender, school, and usage difficulty (i.e., 166 participants experienced usage difficulty)]. No reported significant results below become non-significant when covariates^6^ are included in the model. One non-binary gender participant was not included in the data analysis to allow us to test the potential effect of gender; including this participant did not substantially change the results. Further, 35 participants reported that they had high-quality paint pens (14 in university A and 21 in university B) at the very beginning; since no results are substantively different before and after excluding these 35 participants, we include them when reporting the results below. Details of effects of covariates are included in the Web Appendix.

#### Manipulation Check

A 2×2×2 ANCOVA with participants’ psychological ownership as the dependent measure only revealed a significant main effect of psychological ownership condition [*F*(1, 433)=29.46, *p*<0.001]. As expected, participants in the psychological ownership condition (*M*_psychological own_=3.46, SD=1.99) were more likely to feel that they owned these paint pens than those in the access condition (*M*_access_=2.52, SD=1.71).

#### General Liking

A 2×2×2 ANCOVA with participants’ general liking of the painting activity as the dependent measure revealed no significant effect (*ps*>0.10).

#### Happiness

**Figure 4:**
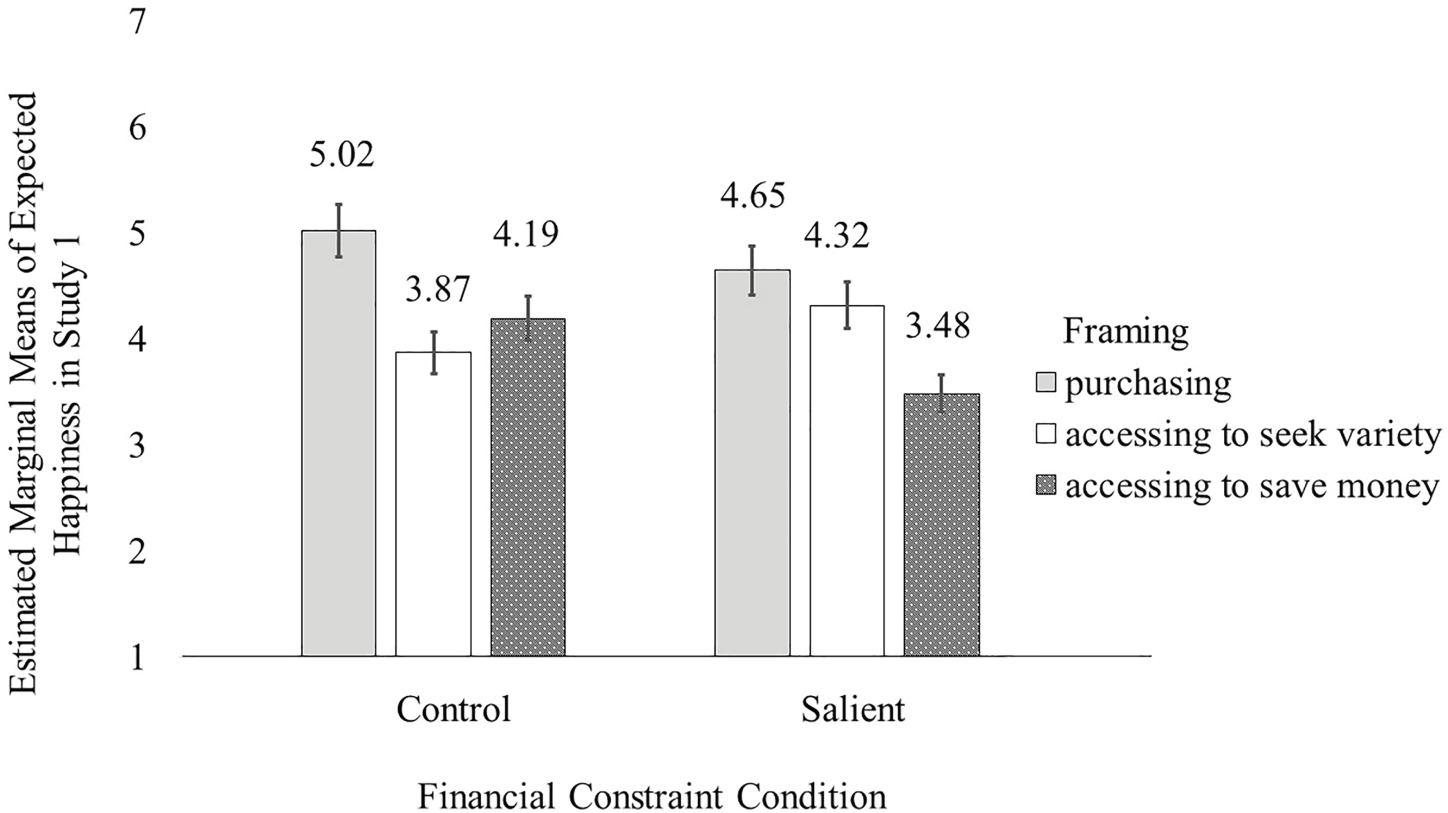
The financial constraints × framing interaction effect on happiness **(A)** and poverty stigma **(B)**, respectively, in Study 4.

A 2×2×2 ANCOVA with happiness as the dependent variable revealed only a significant main effect of framing [*F*(1, 433)=4.22, *p*=0.04], qualified by a constraint × framing interaction that approaches conventional significance [*F*(1, 433)=3.49, *p*=0.06; see Figure 4A]. There was no effect of psychological ownership, nor any two-way or three-way interactions related to psychological ownership (*ps*>0.10).

As expected, participants in the financial constraint salient condition felt less happy when using paint pens from Afford2Paint (*M*_salient+affordability_=3.08, SD=1.17) than from Variety2Paint (*M*_salient+variety_=3.49, SD=1.08; *p*=0.006); this difference was non-significant among those in the control condition (*M*_control+affordability_=3.18, SD=1.16; *M*_control+variety_=3.20, SD=1.09; *p*=0.90).

#### Poverty Stigma

A 2×2×2 ANCOVA with happiness as the dependent variable revealed only a significant main effect of psychological ownership condition [*F*(1, 433)=9.52, *p*=0.002] and a significant financial constraints × framing interaction [*F*(1, 433)=4.43, *p*=0.04; see Figure 4B].

Participants who felt a stronger sense of psychological ownership (*M*_high psychological ownership_=3.32, SD=0.86) felt a lower level of poverty stigma than participants who had a lower sense of psychological ownership (*M*_low psychological ownership_=3.58, SD=0.94). Further, the psychological ownership condition did not interact with either of the other two factors, nor did a three-way interaction emerge (*ps*>0.25).

As expected, participants in the financial constraint salient condition felt a heightened sense of poverty stigma when using paint pens from Afford2Paint (*M*_salient+affordability_=3.60, SD=0.84) as opposed to from Variety2Paint (*M*_salient+variety_=3.36, SD=1.03; *p*=0.04). However, this difference was non-significant among participants for whom financial constraints were not salient (*M*_control+affordability_=3.37, SD=0.91; *M*_control+variety_=3.46, SD=0.86; *p*=0.39).

#### Moderated Mediation

**Figure 5:**
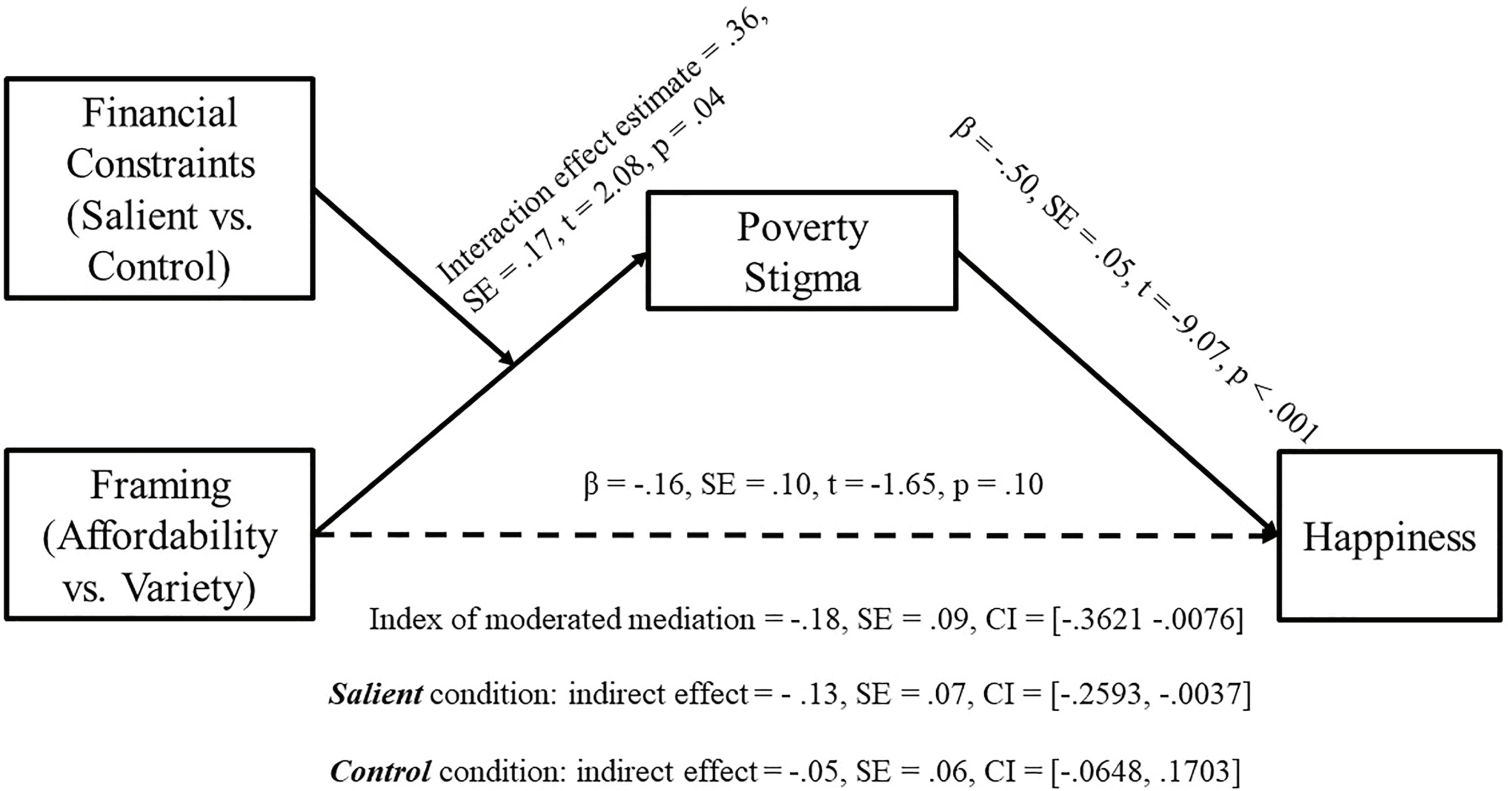
Moderated mediation path diagram (Study 4).

To test whether poverty stigma mediated the effect of framing on financially-constrained individuals’ happiness, we conducted a bias-corrected moderated mediation analysis using PROCESS model 7 (Hayes, 2013) with 20,000 bootstrapped samples (see Figure 5). In this analysis, framing served as the independent variable, financial constraints served as the moderator, poverty stigma served as the mediator, happiness served as the dependent variable, and mode served as the covariate. Supporting our prediction, this analysis indicated significant moderated mediation for happiness (index of moderated mediation=−0.18; 95% CI=[−0.37, −0.01]). This suggests that the framing × financial constraint interaction effect on happiness was mediated by poverty stigma, as the 95% confidence interval of the index of the moderated mediation did not contain zero (Hayes, 2013). In addition, this analysis showed that across the constraints salient condition, participants’ happiness in the affordability framing condition was significantly lower than that in the variety framing (the estimate of the difference between two framing conditions=−0.13, CI=[−0.26, −0.001]). By contrast, across the control condition, participants’ happiness did not differ between the two framing conditions because the 95% CI of the estimate of the difference between the two framing conditions contained zero (estimate=0.05, CI=[−0.06, 0.17]).

#### Other Measures

We checked the effect of framing and financial constraints on other items we measured at the same time as happiness: the perception that participants were smart, intelligent, interesting, and bored. Three-way ANOVAs with each of these items as the dependent variable did not reveal a significant financial constraint × framing interaction effect, nor a three-way interaction. However, there are a significant main effect on smartness and a significant financial constraint × psychological ownership interaction. First, the three-way ANOVA with the feeling of being smart revealed a significant main effect of psychological ownership such that participants in the high psychological ownership condition felt smarter than those in the low psychological ownership condition [*M*_high psychological ownership_=2.40, SD=1.11; *M*_low psychological ownership_=2.21, SD=0.94; *F*(1, 433)=4.19, *p*=0.04]. Second, the three-way ANOVA with feeling intelligent revealed a significant financial constraint × psychological ownership interaction effect [*F*(1, 433)=6.38, *p*=0.01]. Specifically, in the financial constraint salient condition, higher psychological ownership of one’s shared paints pens made one felt more intelligent (*M*_salient+high psychological ownership_=2.49, SD=1.18; *M*_salient+low psychological ownership_=2.08, SD=0.92; *p*=0.003). In the control condition, strengthening psychological ownership did not make one feel more intelligent (*M*_salient+high psychological ownership_=2.26, SD=1.02; *M*_salient+low psychological ownership_=2.35, SD=1.01; *p*=0.56). Both of these findings support the account that strengthening the psychological ownership of shared goods may enhance the appeal of access-based consumption, similar to findings in the previous literature (Bagga et al., 2019; Danckwerts and Kenning, 2019; Fritze et al., 2020). However, unfortunately, strengthening psychological ownership does not appear to financially-constrained individuals alleviate poverty stigma in our sample.

#### Discussion

In Study 4, we replicated the findings of the previous studies in a different product category (i.e., paint pens), given an actual usage experience. We also tested the proposed underlying process, poverty stigma. Though we did not see that participants reported different levels of liking of the painting activity, we did find that when financial constraints were salient, affordability framing vs. variety framing enhanced the level of poverty stigma financially constrained individuals experienced. In turn, this heightened poverty stigma decreased the happiness generated by consumption for these individuals.

We also note that our manipulation of psychological ownership, while creating a main effect on perceptions of poverty stigma, did not interact with either the salience of financial constraints or the framing used to describe the accessed goods. This tells us two things: First, our prior results did not emerge because consumers who accessed products were frustrated with their lack of psychological ownership, *per se*, which may have undermined their happiness. Second, that the benefits of variety-based framing, as opposed to affordability framing, emerge for shared products that are seen as relatively more “owned” as well as those that are seen as more “accessed” shows the robustness of our effect. It does not appear that raising the psychological ownership associated with an accessed good will make an affordability framing acceptable: Financially-constrained individuals may still experience poverty stigma when they access products to save money vs. seek variety – even in cases where they feel they psychologically “own” the shared products.

## General Discussion

Responding to a recent call for research on whether access-based services, or the so-called “sharing economy,” can contribute to consumer well-being,^7^ our research empirically examines framings of access-based consumption that might (or might not) most effectively contribute to financially-constrained consumers’ happiness. We focus on testing the effect of framing in terms of two access-based consumption’ benefits, namely resource affordability and choice variety. Despite the reasons to assume that offering financially-constrained individuals affordable resources vs. choice variety may be more effective in aiding their sense of financial insecurity, our findings suggest that framing access-based consumption in terms of affordability (vs. variety) will trigger a heightened level of poverty stigma, jeopardizing the positive utility of such framing. Across four studies and various product categories, we provide robust evidence that financially-constrained individuals extract less happiness when access-based options are framed in terms of affordability vs. variety. Additionally, we show that this effect is driven by the level of poverty stigma elicited by each type of framing.

We contribute to theory in three ways. First, our research examines consumers’ perception of brand communications in terms of different benefits of access-based consumption. Whereas most prior work on access-based consumption has focused on studying the motives for consumers to join access-based consumption (Ozanne and Ballantine, 2010; Hamari et al., 2016; Schaefers et al., 2016) and the factors that increase their usage intention for access-based services (Lamberton and Rose, 2012; Akbar et al., 2016; Aspara and Wittkowski, 2019; Graul and Brough, 2020), we study how framing access-based consumption in two popular motives, saving money and seeking variety (Lawson et al., 2016; Schaefers et al., 2016; Smith, 2016; Böcker and Meelen, 2017; Cannon et al., 2019), can impact users’ happiness. By delineating the pros and cons of each framing on consumer financial-related identity, we provide a comprehensive framework on how to better frame access-based consumption in order to enhance its contribution to financially-constrained individuals’ happiness in this context.

Interestingly, our work documents a discrepancy between consumers’ motives in using access-based consumption and their happiness from this consumption. Analysts conjecturing about the growth in access-based consumption in the last decade initially anticipated that consumers would be more likely to choose access-based consumption for its ability to offer financial savings and variety (PwC, 2014). When we review more recent work, we see that these two motivations remain visible, particularly in that they correlate with membership in identified segments of access-based consumers (Lawson et al., 2016). However, across studies, we do not find evidence that these two factors change peoples’ decisions to consume *via* access. Rather, we find that financially-constrained consumers simply feel *less happy* when they use such services framed in terms of affordability vs. variety. Thus, it may be that the dissatisfied consumer who has accessed a good will not necessarily recognize why they are not particularly interested in re-consuming *via* the same mode again, even though they retain the same motivations that drew them to access in the first place. As post-consumption happiness is an important factor that drives future consumption (Alba and Williams, 2013), this finding suggests that we may have more to learn about the ways that the factors consumers expect to extract from access-based consumption relate to the experiences they actually have, and the way this relationship affects subsequent choices.

We also contribute to the marketplace stigmatization literature (Chaney et al., 2019; Harmeling et al., 2021) by identifying the potential stigmas in the context of access-based consumption and how these stigmas can impact financially-constrained consumers’ happiness. Little research has considered the identity risk (Blocker et al., 2013; Dalton and Huang, 2014) that financially-constrained consumers may face due to the marketplace stigmas in this context. As shown in this paper, neglecting those stigmas may lead to negative consequences on financially-constrained individuals’ happiness.

Practically, the managerial takeaways from these findings are quite clear. First, our findings highlight the importance of understanding a target consumer’s sense of financial constraints. This subjective sense of lacking enough economic resources may or may not be related to objective financial standing; in our studies, it was fairly easy to manipulate peoples’ perceptions of their financial constraint levels in ways that affected their responses to marketing stimuli. Managers may do well to monitor or proactively shape their target consumers’ sense of financial constraint, such that they can most effectively tailor their communication of access-based opportunities. Second, our findings suggest that companies and non-profit organizations providing access-based goods and services should carefully consider the framing used to promote such options, as the wrong framing may reinforce, rather than weaken, poverty-based stigmatization. Moreover, despite the fact that they almost certainly *do* make consumption more affordable, organizations (e.g., libraries, “Feeding America”) may consider broadcasting the benefits of their access-based services in terms of variety rather than affordability, as doing so will not negatively impact financially-constrained individuals’ usage intention but can make these consumers happier.

Finally, our work has three major limitations, which we consider as avenues for future research. First, building on recent work highlighting the importance of subjective well-being (Diener and Lucas, 2000; Clark et al., 2008), we have focused on happiness as a critical outcome. Given the relationship of happiness to other indicators of well-being (Kahneman et al., 1999), we feel this is an important variable to capture. However, we have not found evidence that this happiness translates into discrete positive (i.e., pride and fulfillment) or negative emotions (i.e., shame, embarrassment, and guilt). We may anticipate that when faced with a low level of happiness and a heightened sense of stigma in the access-based consumption context, financially-constrained people may engage in compensatory consumption behaviors as a self-regulatory strategy to alleviate their sense of financial constraint (Cannon et al., 2019). If this is the case, then savings created by accessing as opposed to purchasing a product may be absorbed in other purchases. If so, the contribution of access-based consumption to society (at least, if not properly framed) might need to be re-evaluated – it may, in fact, activate behaviors that worsen constrained people’s financial position. Besides this, happiness is a short-term consumer well-being measure. It is unknown whether there is a long-term effect of access-based consumption on consumer well-being as a function of different types of framing. Future work may fill in this literature gap by finding a way to track consumer well-being using long-term well-being measures, such as life satisfaction.

Second, we demonstrate how affordability-based (*variety-based*) framing can enhance (*alleviate*) poverty stigma (i.e., people’s self-perception of being poor; Hall et al., 2014) in the context of consumer-to-business access-based consumption. Though we explored the possibility that heightening psychological ownership might mitigate the stigma of accessing for the sake of affordability, we did not observe such an interactive effect. Future studies may explore other ways to mitigate poverty stigma in such a condition. Additionally, we only examined two types of framing within a consumer-to-business model; we selected these framings in part because of their widespread use in the market and their connection to fundamental consumer needs and norms such as economic viability and variety-seeking. Future research can explore how other types of framing based on the benefits in a peer-to-peer model, such as social capital and environmental value (Hawlitschek et al., 2018; Neunhoeffer and Teubner, 2018), may impact consumer well-being.

Third, our study results are based on the sample in the United States, primarily collected immediately prior to and during the COVID-19 pandemic. Cultural differences may impact our findings. First, rent stigma, which has been shown to be prevalent in multiple countries (in United States, Cohen et al., 2009; in United Kingdom, Foye et al., 2018; in Israel, Kricheli-Katz and Posner, 2020), may be weaker in some cultures (Lee and Huang, 2021). Second, our samples in the United States are fairly pro-ownership, in general. This may reflect existing priorities for ownership in the North American market. However, in a more balanced sample, where rent stigma is lower, we may observe different effects. Therefore, future work can focus on studying the role of culture in moderating the effect of framing on financially-constrained individuals’ happiness. Moreover, research also shows that Americans and Indians may be attracted to rent for different reasons – maximized hedonic enjoyment and maximized utilitarian value, respectively (Davidson et al., 2018). Therefore, it might be possible that affordability framing can bring more happiness to financially-constrained consumers in India due to their preference for utilitarian value. Finally, given the barriers created by a pandemic in creating real access-based consumption situations experimentally (as we did in study 4), future research may fruitfully seek additional field replication. Ideally, working together, academics and practitioners can identify ways to frame access-based consumption that brings greater happiness, and with it, well-being, to consumers regardless of financial constraint.

## Data Availability Statement

All study materials and data presented in this paper are available on request to the corresponding author.

## Ethics Statement

The studies involving human participants were reviewed and approved by University of Pittsburgh Institutional Review Board and the University of Pennsylvania Institutional Review Board.

## Author Contributions

This paper is based on YG doctoral dissertation essay under the supervision of CL. YG collected and analyzed the data and wrote the first draft of the manuscript. CL participated in experimental design, supervised analysis, and collaborated on the manuscript’s structure and content. All authors contributed to the article and approved the submitted version.

## Conflict of Interest

The authors declare that the research was conducted in the absence of any commercial or financial relationships that could be construed as a potential conflict of interest.

## Publisher’s Note

All claims expressed in this article are solely those of the authors and do not necessarily represent those of their affiliated organizations, or those of the publisher, the editors and the reviewers. Any product that may be evaluated in this article, or claim that may be made by its manufacturer, is not guaranteed or endorsed by the publisher.

## Footnotes

^1^This is a subjective determination; while the chances of financial constraint are higher among individuals with lower SES or income, high-income people may also feel financially constrained if they have unmet desires (i.e., the “rich but miserable” stereotype, Kay and Jost, 2003), and low-income people may not feel financially-constrained if they have realized their desires (i.e., the “poor but happy” stereotype, Kay and Jost, 2003). As a result, in this paper, instead of using absolute wealth measures (e.g., income), we manipulate participants’ feelings of financial constraint across all studies, such that our theory can be applied to a wider range of consumers (i.e., those who feel financially-constrained, regardless of their present wealth).

^2^Study 1 was launched in March 2019; Study 2 was launched in June 2019; Study 3 was launched in November 2020; and Study 4 was launched in February 2020.

^3^Out of these covariates, only the perceived realism of the study scenario significantly predicted happiness (*b* = 0.42, *SE* = 0.05, *t* = 7.98, *p* < 0.001). Age, gender, income, and annual budget for clothing did not predict happiness.

^4^Out of these covariates, only the perceived realism of the study scenario significantly predicted happiness (*b* = 0.31, *SE* = 0.06, *t* = 5.48, *p* < 0.001). Age, gender, income, car ownership condition, having a valid drivers’ license (or not) did not predict happiness.

^5^Prior research shows that people who are poverty-stigmatized have the following traits (Lindqvist et al., 2017): unreliable (Bullock, 1999), irresponsible and incompetent (Lindqvist et al., 2017), poor and lazy (Cozzarelli et al., 2001).

^6^When predicting happiness: Experiencing usage difficulty has a negative effect on happiness (*b* = −0.32, *SE* = 0.11, *t* = −2.85, *p* = 0.005); gender and school did not significantly predict happiness. When predicting poverty stigma: experiencing usage difficulty significantly increases the sense of poverty stigma (*b* = 0.35, *SE* = 0.09, *t* = 3.97, *p* < 0.001); participants from university B are more likely to experience poverty stigma than those from university A (*b* = 0.25, *SE* = 0.09, *t* = 2.88, *p* = 0.004); and women are less likely than men to experience poverty stigma (*b* = −0.29, *SE* = 0.09, *t* = −3.24, *p* = 0.001).

^7^Frontiers’ call for research related to the topic “Can the Sharing Economy Contribute to Wellbeing? Exploring the Impact of the Sharing Economy on Individual and Collective Wellbeing." Available at: https://www.frontiersin.org/research-topics/21673/can-the-sharing-economy-contribute-to-wellbeing-exploring-the-impact-of-the-sharing-economy-on-indiv
