# Supplementary material for: When Does Sharing Stigmatize? Saving Money (vs. Seeking Variety) Through Access-Based Consumption
Source: Front Psychol. 2021 Nov 18;12:778290. doi: 10.3389/fpsyg.2021.778290 (PMC8637323; doi:10.3389/fpsyg.2021.778290)
Supplement: Supplementary file 1 [file Data_Sheet_1.DOCX]

**Web Appendix:**

**When Does Sharing Stigmatize?**

**Saving Money (vs. Seeking Variety) Through Access-Based Consumption**

List of Content

**1. STUDY 1**

1.1 Other Measures and Results Summary

*1.1.1 Fulfillment*

*1.1.2 Word-of-Mouth tendency*

*1.1.3 Emotions (Pride, Shame, Embarrassment)*

*1.1.4 Rental intention*

*1.1.5 Judgment of Variety-Seeking*

1.2 Robustness Checks

*1.2.1 ANOVA table using full sample*

*1.2.2 ANCOVA table with covariates*

**2. STUDY 2**

2.1 ANOVA table using full sample

2.2 ANCOVA table with covariates

**3. STUDY 3**

3.1 ANOVA table for the reported results in the main text

3.2 ANCOVA table with covariates

**4. STUDY 4**

4.1 ANOVA tables for the reported results in the main text

4.2 ANCOVA tables with covariates

4.3 PROCESS Model 7 Output

4.4 Three-Way ANOVA Tables of Other Measures

**1. STUDY 1**

1.1 Other Measures and Results Summary

*1.1.1 Fulfillment*

*Measure*: “How fulfilled would you feel after doing so to get your clothes?” (scale: 1 = Not fulfilled at all; 7 = Very fulfilled)

*Results*: Two-way ANOVA results revealed only a main effect of framing (F(2, 340) = 9.66, p < .001). The interaction effect was non-significant (p = .31). Post-hoc analysis showed that purchasing clothes (M = 4.66, SD = 1.55) made participants more fulfilled than accessing to save money (M = 3.70, SD = 1.91; p < .001). However, participants felt similarly fulfilled when purchasing clothes and when accessing to seek variety (M = 4.25, SD = 1.79; p = .17).

*1.1.2 Word-of-Mouth tendency*

*Measure*: “If other people knew about the way you obtained your work wardrobe, to what extent would

you feel ____________ ?” (1 = Not at all; 5 = Very)

- Item 1: “like I would want to talk with them about it.”
- Item 2: “Like I would want nobody to know it.” (reverse-coded)

*Results*: The two items were combined to form a word-of-mouth index (*ρ* = .28, *p* < .001). Two-way ANOVA results revealed a main effect framing (F(2, 340) = 14.79, *p* < .001), qualified by a significant two-way interaction (F(2, 340) = 3.21, *p* = .04). However, our focal pairwise comparison was non-significant: Participants had a similar word-of-mouth tendency when they accessed clothes to save money and seek variety (M_salient+affordability_ = 2.32, SD = 1.16; M_salient+variety_ = 2.50, SD = .98; *p* > .99). The only significant pairwise comparisons were: When participants’ financial constraints were made salient, they had a higher word-of-mouth tendency in the purchasing condition (M_salient+purchase_ = 3.37, SD = .90) than the affordability condition and the variety condition, respectively (*ps* < .001).

*1.1.3 Emotions (Pride, Shame, Embarrassment)*

*Measure*: “If other people knew about the way you obtained your work wardrobe, to what extent would

you feel ____________ ?” (1 = Not at all; 5 = Very)

- Ashamed
- Embarrassed
- Proud

*Results*:

*Shame.* Results of the two-way ANOVA with shame as the dependent variable revealed a significant main effect of framing (F(2, 340) = 16.65, *p* < .001) and a marginally significant main effect of financial constraints (F(1, 340) = 3.78, *p* = .05). The interaction effect was non-significant (*p* = .22). Participants felt more ashamed when they accessed clothes regardless of the framing (M_variety_ = 2.34, SD = 1.36; M_affordability_ = 2.42, SD = 1.36; variety vs. affordability: *p* > .99), compared to when they purchased clothes (M_purchase_ = 1.56, SD = .95; *ps* < .001).

*Embarrassment.* Results of the two-way ANOVA with embarrassment as the dependent variable revealed a significant main effect of framing (F(2, 340) = 25.06, *p* < .001) and a significant main effect of financial constraints (F(1, 340) = 5.31, *p* = .02), qualified by a marginally significant interaction effect (F(2, 340) = 2.77, *p* = .06). Participants felt more embarrassed when they accessed clothes regardless of the framing (M_variety_ = 2.53, SD = 1.35; M_affordability_ = 2.64, SD = 1.48; variety vs. affordability: *p* = .79), compared to when they purchased clothes (M_purchase_ = 1.56, SD = .92; *ps* < .001). Our focal comparison was non-significant: When participants’ financial constraints were made salient, they felt similarly embarrassed when they accessed clothes to save money and seek variety (M_salient+affordability_ = 3.02, SD = 1.53; M_salient+variety_ = 2.68, SD = 1.30; *p* = .42).

*Pride.* Results of the two-way ANOVA with pride as the dependent variable revealed only a main effect of framing (F(2, 340) = 10.30, *p* < .001). Participants felt less proud when they accessed clothes regardless of the framing (M_variety_ = 3.25, SD = 1.30; M_affordability_ = 2.44, SD = 1.28; variety vs. affordability: *p* > .99), compared to when they purchased clothes (M_purchase_ = 3.03, SD = 1.19; *ps* < .01).

*1.1.3 Rental intention*

*Measure:* “If you had the choice, would you prefer to obtain your clothes in this scenario by: ______.”

Purchasing.

Renting.

*Results:* In general, 31 participants chose to rent, and 315 participants chose to purchase. The results of a binary logistic regression analysis (coding: renting = 0, purchasing = 1) revealed no significant effect (see the table below).

| Variables in the Equation | | | | | | | |
| --- | --- | --- | --- | --- | --- | --- | --- |
|  | | B | S.E. | Wald | df | Sig. | Exp(B) |
| Step 1^a^ | constraint(1) | -17.706 | 5862.764 | 0.000 | 1.000 | 0.998 | 0.000 |
|  | framing |  |  | 0.195 | 2.000 | 0.907 |  |
|  | framing(1) | -19.485 | 5862.764 | 0.000 | 1.000 | 0.997 | 0.000 |
|  | framing(2) | -19.239 | 5862.764 | 0.000 | 1.000 | 0.997 | 0.000 |
|  | constraint * framing |  |  | 0.020 | 2.000 | 0.990 |  |
|  | constraint(1) by framing(1) | 17.869 | 5862.764 | 0.000 | 1.000 | 0.998 | 57600987.528 |
|  | constraint(1) by framing(2) | 17.983 | 5862.764 | 0.000 | 1.000 | 0.998 | 64584749.416 |
|  | Constant | 21.203 | 5862.764 | 0.000 | 1.000 | 0.997 | 1615477575.145 |
| a. Variable(s) entered on step 1: constraint, framing, constraint * framing . | | | | | | | |

*1.1.4 Judgment of Variety-Seeking*

*Measure:* “Please give your opinion about the following statements based on your current daily consumption (e.g., living and shopping habits). To what extent do you agree or disagree with the following statements?”

- Item 1: Seeking variety in daily consumption is beyond my current means.
- Item 2: It's not a good idea for me to make it a goal to have high variety in my consumption.
- Item 3: It is inappropriate for people to prioritize variety in their daily consumption.
- Item 4: It's a virtue to seek a lot of variety in one's consumption. [reverse-coded]
- Item 5: People who don't pay for variety are better people than those who do.
- Item 6: People who have tight budgets should be content without worrying about variety in their consumption.
- Item 7: People who seek a lot of variety in their consumption are often judged harshly by others.

“Now we will ask you how OTHERS tend to judge people when they consume a lot of variety. This may or may not be the way you see things - we're just interested in how you think most people think or feel.”

- Item 8: People who prioritize variety in their consumption are seen as irresponsible by those around them.
- Item 9: People who consume a lot of variety are often seen as better people than those who consume the same thing over and over. [reverse-coded]
- Item 10: Consuming variety is seen as a sign that someone is an interesting person. [reverse-coded]

*Results:* We created the above items. For the first set of statements, all items were entered into a factor analyses and did not load to one factor (see below). However, we combined all seven items into one index (*α* = .62) because the financial constraints x framing interaction was non-significant regardless of whether one index or each item were entered as the dependent variable.

| Rotated Component Matrix | | | | | | |
| --- | --- | --- | --- | --- | --- | --- |
|  | Raw | | | Rescaled | | |
|  | Component | | | Component | | |
|  | 1 | 2 | 3 | 1 | 2 | 3 |
| Seeking variety in daily consumption is beyond my current means. | 0.196 | **1.004** | -0.077 | 0.171 | 0.876 | -0.068 |
| It's not a good idea for me to make it a goal to have high variety in my consumption. | 0.014 | **0.962** | 0.342 | 0.012 | 0.835 | 0.297 |
| It is inappropriate for people to prioritize variety in their daily consumption. | 0.464 | 0.282 | **0.702** | 0.419 | 0.255 | 0.634 |
| It's a virtue to seek a lot of variety in one's consumption. [reverse-coded] | -0.694 | 0.041 | 0.356 | -0.678 | 0.040 | 0.348 |
| People who don't pay for variety are better people than those who do. | **0.753** | 0.111 | 0.394 | 0.684 | 0.101 | 0.358 |
| People who have tight budgets should be content without worrying about variety in their consumption. | **-**0.002 | 0.044 | **0.958** | -0.002 | 0.038 | 0.830 |
| People who seek a lot of variety in their consumption are often judged harshly by others. | **0.831** | 0.199 | 0.237 | 0.738 | 0.176 | 0.210 |
| Extraction Method: Principal Component Analysis.   Rotation Method: Varimax with Kaiser Normalization. | | | | | | |
| a. Rotation converged in 5 iterations. | | | | | | |

For the second set of statements, the inter-item reliability is very low (*α* = -.006) and cannot be combined to one index even though all three items loaded into one factor. So, conducted three ANOVA analyses with each of these items as the dependent measure. Again, results revealed no significant financial constraints x framing interaction effect.

To sum up, our results suggest that participants do not stigmatize those who seek variety in the access-based consumption context.

1.2 Robustness Checks

*1.2.1 ANOVA table using full sample:*

| Two-way ANOVA | *SS* | *DF* | *MS* | *F* | *p-value* | *Partial η^2^* | *Observed Power* |
| --- | --- | --- | --- | --- | --- | --- | --- |
| Intercept | 6847.436 | 1 | 6847.436 | 2565.619 | < .001 | .876 | 1.000 |
| Financial Constraints | 2.803 | 1 | 2.803 | 1.050 | .306 | .003 | .176 |
| Framing | 50.970 | 2 | 25.485 | 9.549 | < .001 | .050 | .980 |
| Financial Constraints * Framing | 15.897 | 2 | 7.948 | 2.978 | .052 | .016 | .577 |
| Error | 971.488 | 364 | 2.669 |  |  |  |  |

*1.2.2 ANCOVA Table with covariates:*

| Two-way ANCOVA | SS | DF | MS | F | p-value | Partial η2 | Observed Power |
| --- | --- | --- | --- | --- | --- | --- | --- |
| Intercept | 85.019 | 1 | 85.019 | 38.590 | 0.000 | 0.098 | 1.000 |
| Income | 2.037 | 1 | 2.037 | 0.924 | 0.337 | 0.003 | 0.160 |
| Age | 3.628 | 1 | 3.628 | 1.647 | 0.200 | 0.005 | 0.249 |
| Gender | 4.190 | 1 | 4.190 | 1.902 | 0.169 | 0.005 | 0.280 |
| Annual Clothing Budget | 3.689 | 1 | 3.689 | 1.674 | 0.197 | 0.005 | 0.252 |
| Perceived Realism of the Scenario | 140.449 | 1 | 140.449 | 63.749 | < .001 | 0.152 | 1.000 |
| Financial Constraints | 2.083 | 1 | 2.083 | 0.946 | 0.332 | 0.003 | 0.163 |
| Framing | 26.143 | 2 | 13.072 | 5.933 | 0.003 | 0.032 | 0.877 |
| Financial Constraints * Framing | 18.310 | 2 | 9.155 | 4.155 | 0.016 | 0.023 | 0.731 |
| Error | 782.121 | 355 | 2.203 |  |  |  |  |

(Note: Four participants who did not provide their gender were excluded from the above analysis).

**2. STUDY 2**

2.1 ANOVA table using full sample

| Two-way ANOVA | *SS* | *DF* | *MS* | *F* | *p-value* | *Partial η^2^* | *Observed Power* |
| --- | --- | --- | --- | --- | --- | --- | --- |
| Intercept | 3745.895 | 1 | 3745.895 | 3853.797 | < .001 | 0.928 | 1.000 |
| Financial Constraints | 10.988 | 1 | 10.988 | 11.305 | 0.001 | 0.036 | 0.918 |
| Framing | 1.337 | 1 | 1.337 | 1.376 | 0.242 | 0.005 | 0.215 |
| Financial Constraints * Framing | 4.816 | 1 | 4.816 | 4.954 | 0.027 | 0.016 | 0.602 |
| Error | 292.572 | 301 | 0.972 |  |  |  |  |

2.2 ANCOVA table with covariates

| Two-way ANCOVA | *SS* | *DF* | *MS* | *F* | *p-value* | *Partial η^2^* | *Observed Power* |
| --- | --- | --- | --- | --- | --- | --- | --- |
| Intercept | 15.579 | 1 | 15.579 | 18.447 | 0.000 | 0.059 | 0.990 |
| *Gender* | 0.107 | 1 | 0.107 | 0.127 | 0.722 | 0.000 | 0.065 |
| *Income* | 1.671 | 1 | 1.671 | 1.979 | 0.161 | 0.007 | 0.289 |
| *Age* | 0.589 | 1 | 0.589 | 0.698 | 0.404 | 0.002 | 0.132 |
| *Car Ownership* | 2.951 | 1 | 2.951 | 3.495 | 0.063 | 0.012 | 0.462 |
| *Perceived realism of the scenario* | 25.313 | 1 | 25.313 | 29.973 | < .001 | 0.093 | 1.000 |
| *Having a valid driver’s license* | 0.587 | 1 | 0.587 | 0.695 | 0.405 | 0.002 | 0.132 |
| Financial Constraints | 6.627 | 1 | 6.627 | 7.847 | 0.005 | 0.026 | 0.797 |
| Framing | 1.018 | 1 | 1.018 | 1.205 | 0.273 | 0.004 | 0.194 |
| Financial Constraints * Framing | 2.606 | 1 | 2.606 | 3.086 | 0.080 | 0.010 | 0.417 |
| Error | 248.289 | 294 | 0.845 |  |  |  |  |

**3. STUDY 3**

3.1 ANOVA table for the reported results in the main text

| Two-way ANOVA | *SS* | *DF* | *MS* | *F* | *p-value* | *Partial η^2^* | *Observed Power* |
| --- | --- | --- | --- | --- | --- | --- | --- |
| Intercept | 10077.374 | 1 | 10077.374 | 3736.659 | < .001 | 0.824 | 1.000 |
| Financial Constraints | 150.470 | 1 | 150.470 | 55.794 | < .001 | 0.065 | 1.000 |
| Framing | 167.195 | 3 | 55.732 | 20.665 | < .001 | 0.072 | 1.000 |
| Financial Constraints * Framing | 52.114 | 3 | 17.371 | 6.441 | < .001 | 0.024 | 0.970 |
| Error | 2152.122 | 798 | 2.697 |  |  |  |  |

3.2 ANCOVA table with covariates

| Two-way ANCOVA | *SS* | *DF* | *MS* | *F* | *p-value* | *Partial η^2^* | *Observed Power* |
| --- | --- | --- | --- | --- | --- | --- | --- |
| Intercept | 70.393 | 1 | 70.393 | 30.268 | < .001 | 0.037 | 1.000 |
| *Gender* | 0.346 | 1 | 0.346 | 0.149 | 0.700 | 0.000 | 0.067 |
| *Income* | 2.077 | 1 | 2.077 | 0.893 | 0.345 | 0.001 | 0.157 |
| *Age* | 6.394 | 1 | 6.394 | 2.749 | 0.098 | 0.003 | 0.381 |
| *Had Rented Clothing* | 0.053 | 1 | 0.053 | 0.023 | 0.880 | 0.000 | 0.053 |
| *Annual Clothing Budget* | 14.577 | 1 | 14.577 | 6.268 | 0.012 | 0.008 | 0.706 |
| *Perceived Realism of the Scenario* | 266.148 | 1 | 266.148 | 114.438 | < .001 | 0.127 | 1.000 |
| Financial Constraints | 149.973 | 1 | 149.973 | 64.485 | < .001 | 0.075 | 1.000 |
| Framing | 140.615 | 3 | 46.872 | 20.154 | < .001 | 0.071 | 1.000 |
| Financial Constraints * Framing | 49.801 | 3 | 16.600 | 7.138 | < .001 | 0.026 | 0.982 |
| Error | 1837.294 | 790 | 2.326 |  |  |  |  |

**4. STUDY 4**

4.1 ANOVA tables for the reported results in the main text

**DV = Happiness**

| Three-way ANOVA | *SS* | *DF* | *MS* | *F* | *p-value* | *Partial η^2^* | *Observed Power* |
| --- | --- | --- | --- | --- | --- | --- | --- |
| Intercept | 4622.527 | 1 | 4622.527 | 3624.895 | < .001 | 0.893 | 1.000 |
| Financial Constraints | 1.349 | 1 | 1.349 | 1.058 | 0.304 | 0.002 | 0.177 |
| Psychological Ownership | 2.266 | 1 | 2.266 | 1.777 | 0.183 | 0.004 | 0.265 |
| Framing | 4.947 | 1 | 4.947 | 3.880 | 0.050 | 0.009 | 0.502 |
| Financial Constraints * Psychological Ownership | 1.507 | 1 | 1.507 | 1.182 | 0.278 | 0.003 | 0.192 |
| Financial Constraints * Framing | 4.055 | 1 | 4.055 | 3.180 | 0.075 | 0.007 | 0.428 |
| Psychological Ownership * Framing | 0.402 | 1 | 0.402 | 0.316 | 0.575 | 0.001 | 0.087 |
| Financial Constraints * Psychological Ownership * Framing | 0.085 | 1 | 0.085 | 0.067 | 0.796 | 0.000 | 0.058 |
| Error | 553.444 | 434 | 1.275 |  |  |  |  |

**DV = Poverty Stigma**

| Three-way ANOVA | *SS* | *DF* | *MS* | *F* | *p-value* | *Partial η^2^* | *Observed Power* |
| --- | --- | --- | --- | --- | --- | --- | --- |
| Intercept | 5223.503 | 1 | 5223.503 | 6441.623 | < .001 | 0.937 | 1.000 |
| Financial Constraints | 0.279 | 1 | 0.279 | 0.344 | 0.558 | 0.001 | 0.090 |
| Psychological Ownership | 7.791 | 1 | 7.791 | 9.608 | 0.002 | 0.022 | 0.871 |
| Framing | 0.652 | 1 | 0.652 | 0.805 | 0.370 | 0.002 | 0.146 |
| Financial Constraints * Psychological Ownership | 0.004 | 1 | 0.004 | 0.005 | 0.946 | 0.000 | 0.051 |
| Financial Constraints * Framing | 3.633 | 1 | 3.633 | 4.481 | 0.035 | 0.010 | 0.560 |
| Psychological Ownership * Framing | 0.920 | 1 | 0.920 | 1.134 | 0.287 | 0.003 | 0.186 |
| Financial Constraints * Psychological Ownership * Framing | 0.760 | 1 | 0.760 | 0.937 | 0.334 | 0.002 | 0.162 |
| Error | 351.930 | 434 | 0.811 |  |  |  |  |

4.2 ANCOVA tables with covariates

**DV = Happiness**

| Three-way ANCOVA | SS | DF | MS | F | p-value | Partial η2 | Observed Power |
| --- | --- | --- | --- | --- | --- | --- | --- |
| Intercept | 400.770 | 1 | 400.770 | 320.643 | < .001 | 0.427 | 1.000 |
| Experienced Usage Difficulty | 10.126 | 1 | 10.126 | 8.102 | 0.005 | 0.018 | 0.811 |
| School | 0.027 | 1 | 0.027 | 0.021 | 0.884 | 0.000 | 0.052 |
| Gender | 2.299 | 1 | 2.299 | 1.840 | 0.176 | 0.004 | 0.272 |
| Financial Constraints | 1.499 | 1 | 1.499 | 1.199 | 0.274 | 0.003 | 0.194 |
| Psychological Ownership | 2.739 | 1 | 2.739 | 2.192 | 0.140 | 0.005 | 0.315 |
| Framing | 5.512 | 1 | 5.512 | 4.410 | 0.036 | 0.010 | 0.554 |
| Financial Constraints * Psychological Ownership | 0.909 | 1 | 0.909 | 0.727 | 0.394 | 0.002 | 0.136 |
| Financial Constraints * Framing | 4.669 | 1 | 4.669 | 3.735 | 0.054 | 0.009 | 0.487 |
| Psychological Ownership * Framing | 0.084 | 1 | 0.084 | 0.067 | 0.796 | 0.000 | 0.058 |
| Financial Constraints * Psychological Ownership * Framing | 2.648E-05 | 1 | 2.648E-05 | 0.000 | 0.996 | 0.000 | 0.050 |
| Error | 537.455 | 430 | 1.250 |  |  |  |  |

(Note: one non-binary gender participant was excluded from the above anlysis to allow us to test if there is a gender effect.)

**DV = Poverty Stigma**

| Three-way ANCOVA | *SS* | *DF* | *MS* | *F* | *p-value* | *Partial η^2^* | *Observed Power* |
| --- | --- | --- | --- | --- | --- | --- | --- |
| Intercept | 377.303 | 1 | 377.303 | 491.484 | 0.000 | 0.533 | 1.000 |
| *Experienced Usage Difficulty* | 12.090 | 1 | 12.090 | 15.749 | 0.000 | 0.035 | 0.977 |
| *School* | 6.350 | 1 | 6.350 | 8.271 | 0.004 | 0.019 | 0.818 |
| *Gender* | 8.040 | 1 | 8.040 | 10.473 | 0.001 | 0.024 | 0.898 |
| Financial Constraints | 0.095 | 1 | 0.095 | 0.123 | 0.726 | 0.000 | 0.064 |
| Psychological Ownership | 8.158 | 1 | 8.158 | 10.627 | 0.001 | 0.024 | 0.902 |
| Framing | 0.863 | 1 | 0.863 | 1.124 | 0.290 | 0.003 | 0.185 |
| Financial Constraints * Psychological Ownership | 0.021 | 1 | 0.021 | 0.027 | 0.869 | 0.000 | 0.053 |
| Financial Constraints * Framing | 3.650 | 1 | 3.650 | 4.754 | 0.030 | 0.011 | 0.585 |
| Psychological Ownership * Framing | 0.487 | 1 | 0.487 | 0.635 | 0.426 | 0.001 | 0.125 |
| Financial Constraints * Psychological Ownership * Framing | 0.190 | 1 | 0.190 | 0.248 | 0.619 | 0.001 | 0.079 |
| Error | 330.103 | 430 | 0.768 |  |  |  |  |

(Note: one non-binary gender participant was excluded from the above anlysis to allow us to test if there is a gender effect.)

4.3 RPOCESS Model 7 Output

Run MATRIX procedure:

**************** PROCESS Procedure for SPSS Version 3.5.3 ****************

Written by Andrew F. Hayes, Ph.D. www.afhayes.com

Documentation available in Hayes (2018). www.guilford.com/p/hayes3

**************************************************************************

**Model : 7**

**Y : Happiness (abbr: hindex)**

**X : Framing (as: framing)**

**M : Poverty Stigma (abbr: ps)**

**W : Financial Constraints (abbr: fc)**

Covariates:

**Psychological Ownership** **(abbr: mode)**

Sample

Size: 442

**************************************************************************

OUTCOME VARIABLE:

ps

Model Summary

R R-sq MSE F df1 df2 p

.1795 .0322 .8092 3.6376 4.0000 437.0000 .0063

Model

coeff se t p LLCI ULCI

constant 3.6041 .0953 37.8374 .0000 3.4169 3.7913

framing -.1046 .1212 -.8627 .3888 -.3428 .1337

fc -.1243 .1210 -1.0267 .3051 -.3621 .1136

Int_1 .3571 .1715 2.0828 .0378 .0201 .6941

mode -.2626 .0857 -3.0642 .0023 -.4310 -.0942

Product terms key:

Int_1 : framing x fc

Covariance matrix of regression parameter estimates:

constant framing fc Int_1 mode

constant .0091 -.0071 -.0073 .0073 -.0039

framing -.0071 .0147 .0070 -.0147 .0003

fc -.0073 .0070 .0146 -.0147 .0005

Int_1 .0073 -.0147 -.0147 .0294 -.0006

mode -.0039 .0003 .0005 -.0006 .0073

Test(s) of highest order unconditional interaction(s):

R2-chng F df1 df2 p

X*W .0096 4.3381 1.0000 437.0000 .0378

----------

Focal predict: framing (X)

Mod var: fc (W)

Conditional effects of the focal predictor at values of the moderator(s):

fc Effect se t p LLCI ULCI

.0000 -.1046 .1212 -.8627 .3888 -.3428 .1337

1.0000 .2526 .1212 2.0843 .0377 .0144 .4907

Data for visualizing the conditional effect of the focal predictor:

Paste text below into a SPSS syntax window and execute to produce plot.

DATA LIST FREE/

framing fc ps .

BEGIN DATA.

.0000 .0000 3.4728

1.0000 .0000 3.3683

.0000 1.0000 3.3486

1.0000 1.0000 3.6011

END DATA.

GRAPH/SCATTERPLOT=

framing WITH ps BY fc .

**************************************************************************

OUTCOME VARIABLE:

hindex

Model Summary

R R-sq MSE F df1 df2 p

.4092 .1675 1.0778 29.3712 3.0000 438.0000 .0000

Model

coeff se t p LLCI ULCI

constant 5.0332 .2124 23.6946 .0000 4.6157 5.4507

framing -.1634 .0989 -1.6532 .0990 -.3577 .0309

ps -.4978 .0549 -9.0656 .0000 -.6057 -.3899

mode .0033 .0998 .0329 .9738 -.1928 .1994

Covariance matrix of regression parameter estimates:

constant framing prev mode

constant .0451 -.0040 -.0107 -.0076

framing -.0040 .0098 -.0002 -.0001

ps -.0107 -.0002 .0030 .0008

mode -.0076 -.0001 .0008 .0100

****************** DIRECT AND INDIRECT EFFECTS OF X ON Y *****************

Direct effect of X on Y

Effect se t p LLCI ULCI

-.1634 .0989 -1.6532 .0990 -.3577 .0309

Conditional indirect effects of X on Y:

INDIRECT EFFECT:

framing -> ps -> hindex

fc Effect BootSE BootLLCI BootULCI

.0000 .0521 .0597 -.0648 .1703

1.0000 -.1257 .0653 -.2593 -.0037

**(Coding: fc = 0, control condition; fc = 1, salient financial constraints condition)**

Index of moderated mediation (difference between conditional indirect effects):

Index BootSE BootLLCI BootULCI

fc -.1778 .0899 -.3621 -.0076

Pairwise contrasts between conditional indirect effects (Effect1 minus Effect2)

Effect1 Effect2 Contrast BootSE BootLLCI BootULCI

-.1257 .0521 -.1778 .0899 -.3621 -.0076

---

*********** BOOTSTRAP RESULTS FOR REGRESSION MODEL PARAMETERS ************

OUTCOME VARIABLE:

ps

Coeff BootMean BootSE BootLLCI BootULCI

constant 3.6041 3.6030 .0936 3.4212 3.7887

framing -.1046 -.1034 .1184 -.3354 .1294

fc -.1243 -.1219 .1255 -.3683 .1213

Int_1 .3571 .3550 .1719 .0168 .6908

mode -.2626 -.2623 .0859 -.4309 -.0952

----------

OUTCOME VARIABLE:

hindex

Coeff BootMean BootSE BootLLCI BootULCI

constant 5.0332 5.0400 .2183 4.6105 5.4690

framing -.1634 -.1631 .0990 -.3593 .0282

ps -.4978 -.4997 .0578 -.6131 -.3861

mode .0033 .0022 .1012 -.1960 .2024

*********************** ANALYSIS NOTES AND ERRORS ************************

Level of confidence for all confidence intervals in output:

95.0000

Number of bootstrap samples for percentile bootstrap confidence intervals:

20000

------ END MATRIX -----

4.4 Three-Way ANOVA Tables of Other Measures

**DV = Feeling smart**

| Three-way ANOVA | *SS* | *DF* | *MS* | *F* | *p-value* | *Partial η^2^* | *Observed Power* |
| --- | --- | --- | --- | --- | --- | --- | --- |
| Intercept | 2344.927 | 1 | 2344.927 | 2221.661 | 0.000 | 0.837 | 1.000 |
| Financial Constraints | 0.069 | 1 | 0.069 | 0.065 | 0.798 | 0.000 | 0.057 |
| Psychological Ownership | 4.422 | 1 | 4.422 | 4.190 | 0.041 | 0.010 | 0.533 |
| Framing | 1.297 | 1 | 1.297 | 1.229 | 0.268 | 0.003 | 0.198 |
| Financial Constraints * Psychological Ownership | 3.716 | 1 | 3.716 | 3.521 | 0.061 | 0.008 | 0.465 |
| Financial Constraints * Framing | 3.456 | 1 | 3.456 | 3.274 | 0.071 | 0.008 | 0.439 |
| Psychological Ownership * Framing | 0.083 | 1 | 0.083 | 0.079 | 0.779 | 0.000 | 0.059 |
| Financial Constraints * Psychological Ownership * Framing | 0.175 | 1 | 0.175 | 0.166 | 0.684 | 0.000 | 0.069 |
| Error | 457.024 | 433 | 1.055 |  |  |  |  |

**DV = Feeling bored**

| Three-way ANOVA | *SS* | *DF* | *MS* | *F* | *p-value* | *Partial η^2^* | *Observed Power* |
| --- | --- | --- | --- | --- | --- | --- | --- |
| Intercept | 2275.285 | 1 | 2275.285 | 1590.914 | 0.000 | 0.786 | 0.235 |
| Financial Constraints | 2.194 | 1 | 2.194 | 1.534 | 0.216 | 0.004 | 0.074 |
| Psychological Ownership | 0.299 | 1 | 0.299 | 0.209 | 0.648 | 0.000 | 0.073 |
| Framing | 0.283 | 1 | 0.283 | 0.198 | 0.657 | 0.000 | 0.062 |
| Financial Constraints * Psychological Ownership | 0.151 | 1 | 0.151 | 0.106 | 0.745 | 0.000 | 0.260 |
| Financial Constraints * Framing | 2.486 | 1 | 2.486 | 1.738 | 0.188 | 0.004 | 0.192 |
| Psychological Ownership * Framing | 1.691 | 1 | 1.691 | 1.183 | 0.277 | 0.003 | 0.057 |
| Financial Constraints * Psychological Ownership * Framing | 0.084 | 1 | 0.084 | 0.059 | 0.809 | 0.000 | 0.235 |
| Error | 619.266 | 433 | 1.430 |  |  |  |  |

**DV = Feeling intelligent**

| Three-way ANOVA | *SS* | *DF* | *MS* | *F* | *p-value* | *Partial η^2^* | *Observed Power* |
| --- | --- | --- | --- | --- | --- | --- | --- |
| Intercept | 2314.457 | 1 | 2314.457 | 2183.164 | 0.000 | 0.834 | 1.000 |
| Financial Constraints | 0.041 | 1 | 0.041 | 0.038 | 0.845 | 0.000 | 0.054 |
| Psychological Ownership | 3.061 | 1 | 3.061 | 2.887 | 0.090 | 0.007 | 0.396 |
| Framing | 0.197 | 1 | 0.197 | 0.186 | 0.667 | 0.000 | 0.071 |
| Financial Constraints * Psychological Ownership | 6.763 | 1 | 6.763 | 6.379 | 0.012 | 0.015 | 0.712 |
| Financial Constraints * Framing | 1.978 | 1 | 1.978 | 1.866 | 0.173 | 0.004 | 0.276 |
| Psychological Ownership * Framing | 0.113 | 1 | 0.113 | 0.106 | 0.745 | 0.000 | 0.062 |
| Financial Constraints * Psychological Ownership * Framing | 0.020 | 1 | 0.020 | 0.018 | 0.892 | 0.000 | 0.052 |
| Error | 459.040 | 433 | 1.060 |  |  |  |  |

**DV = Feeling interesting**

| Three-way ANOVA | *SS* | *DF* | *MS* | *F* | *p-value* | *Partial η^2^* | *Observed Power* |
| --- | --- | --- | --- | --- | --- | --- | --- |
| Intercept | 4096.141 | 1 | 4096.141 | 2909.387 | 0.000 | 0.870 | 1.000 |
| Financial Constraints | 0.250 | 1 | 0.250 | 0.177 | 0.674 | 0.000 | 0.070 |
| Psychological Ownership | 3.523 | 1 | 3.523 | 2.502 | 0.114 | 0.006 | 0.352 |
| Framing | 2.056 | 1 | 2.056 | 1.460 | 0.228 | 0.003 | 0.226 |
| Financial Constraints * Psychological Ownership | 4.069 | 1 | 4.069 | 2.890 | 0.090 | 0.007 | 0.396 |
| Financial Constraints * Framing | 1.904 | 1 | 1.904 | 1.352 | 0.246 | 0.003 | 0.213 |
| Psychological Ownership * Framing | 0.106 | 1 | 0.106 | 0.075 | 0.784 | 0.000 | 0.059 |
| Financial Constraints * Psychological Ownership * Framing | 0.016 | 1 | 0.016 | 0.012 | 0.914 | 0.000 | 0.051 |
| Error | 609.623 | 433 | 1.408 |  |  |  |  |
